# On the Boundary of Exploratory Genomics and Translation in Sequential Glioblastoma

**DOI:** 10.3390/ijms25147564

**Published:** 2024-07-10

**Authors:** Marton Tompa, Bence Galik, Peter Urban, Bela Istvan Kajtar, Zoltan Kraboth, Attila Gyenesei, Attila Miseta, Bernadette Kalman

**Affiliations:** 1 Szentagothai Research Center, University of Pecs, 20. Ifjusag Street, 7624 Pecs, Hungary; galik.bence@pte.hu (B.G.); urban.peter@pte.hu (P.U.); gyenesei.attila@pte.hu (A.G.); 2 Department of Molecular Medicine, Markusovszky University Teaching Hospital, 5. Markusovszky Street, 9700 Szombathely, Hungary; 3 Department of Pathology, School of Medicine, University of Pecs, 12. Szigeti Street, 7624 Pecs, Hungary; kajtar.bela@pte.hu (B.I.K.); kraboth.zoltan@pte.hu (Z.K.); 4 Office of the Dean, School of Medicine, University of Pecs, 20. Ifjusag Street, 7624 Pecs, Hungary; attila.miseta@aok.pte.hu

**Keywords:** glioblastoma, whole exome sequencing, deep bioinformatics, clinical utility

## Abstract

OMICS methods brought significant advancements to the understanding of tumor cell biology, which transformed the treatment and prognosis of several cancers. Clinical practice and outcomes, however, changed significantly less in the case of glioblastoma (GBM). In this study, we aimed to assess the utility of whole exome (WES) sequencing in the clinical setting. Ten pairs of formalin-fixed, paraffin-embedded (FFPE) GBM specimens were obtained at onset (GBM-P) and at recurrence (GBM-R). Histopathological and molecular features of all samples supported the diagnosis of GBM based on WHO CNS5. WES data were filtered, applying a strict and custom-made pipeline, and occurrence of oncogenic and likely oncogenic variants in GBM-P, GBM-R or both were identified by using the VarSeq program version 2.5.0 (Golden Helix, Inc.). Characteristics and recurrence of the variants were analyzed in our own cohort and were also compared to those available in the COSMIC database. The lists of oncogenic and likely oncogenic variants corresponded to those identified in other studies. The average number of these variants were 4 and 5 out of all detected 24 and 34 variants in GBM-P and GBM-R samples, respectively. On average, one shared oncogenic/likely oncogenic variant was found in the pairs. We assessed the identified variants’ therapeutic significance, also taking into consideration the guidelines by the Association for Molecular Pathology (AMP). Our data support that a thorough WES analysis is suitable for identifying oncogenic and likely oncogenic variants in an individual clinical sample or a small cohort of FFPE glioma specimens, which concur with those of comprehensive research studies. Such analyses also allow us to monitor molecular dynamics of sequential GBM. In addition, careful evaluation of data according to the AMP guideline reveal that though therapeutic applicability of the variants is generally limited in the clinic, such information may be valuable in selected cases, and can support innovative preclinical and clinical trials.

## 1. Introduction

Gliomas are the most common tumors of the central nervous system (CNS) and are traditionally subdivided based on histological grades I–IV, where grade IV glioma represents the most aggressive subtype, glioblastoma (GBM). During the last two decades, comprehensive sequencing of genomic DNA from solid tumors opened a new window for a better understanding of cancer cell biology by identifying characteristic somatic mutations that drive occurrence and progression in various histological entities, including GBM [1,2]. The fourth and fifth editions of the World Health Organization’s (WHO) classification of tumors of the CNS (WHO CNS4 and 5) integrated the most characteristic genomic and genetic changes into the routine histopathological work up [3,4].

While The Cancer Genome Atlas (TCGA) network mostly focused their OMICS analyses on gliomas at onset and proposed molecular subtypes of GBM [1,5,6], the Glioma Longitudinal Analysis Consortium (GLASS) carried out comprehensive genomic and epigenomic analyses to catalogue longitudinal molecular changes [7]. Wang et al. [8] also investigated genomic and transcriptomic data of longitudinal GBM and found that almost two thirds of patients experienced expression-based subtype changes over time. From the branching patterns and the evolutionary rates, the authors inferred that the relapse-associated clone existed years prior to the clinical diagnosis. They also estimated that 15% of tumors may have hypermutation at recurrence in highly expressed genes [8]. Neilsen et al. [9] established that deletions frequently occurred in *CDKN2 A* and *CDKN2 B*, activating mutations or amplifications were common in *EGFR*, and mutations were also frequent in *TERT* and *PI3 K* at both time points. However, the amount of the variants significantly varied in samples, suggesting clonal evolution over time [9]. Barthel et al. [10] noted that driver genes found at onset were typically retained at recurrence. Treatments using alkylating agents led to hypermutator phenotypes but at different rates among glioma subtypes. The study established that the strongest selective pressure affects glioma initiation and early development, while therapies affect the molecular profiles in a stochastic manner [10]. Deep whole genome sequencing by Körber et al. [11] revealed that de novo GBMs commonly carry chromosome 7 gain, 9 p loss or 10 loss early during tumor initiation, while *TERT* promoter mutation may arise later in association with rapid growth [11].

Using a customized enrichment/hybrid capture-based next-generation sequencing (NGS) gene panel, Sahm et al. [12] were able to identify potential treatment targets in various brain tumors using samples in routine neuropathology [12]. In longitudinal whole exome and whole genome analyses of tumors from a proband and a large cohort, Erson-Omay et al. [13] aimed to use genomics-guided personalized treatment to extend the survival of the proband with GBM [13]. Studying common drug targets in primary and recurrent GBM specimens, Schäfer et al. [14] found profound changes in the expressions of 9 out of 10 investigated molecules, with the only exception being *FGFR1*. This heterogeneity during the course of disease suggests that the molecular treatment design needs to be adjusted and personalized over time [14].

Cho et al. [15] used targeted NGS from primary and recurrent GBM to evaluate the mutational status of six DNA repair-related genes. The authors found both germline and somatic mutations, most frequently in *MSH6* and *POLE*. The presence of *MGMT* (O^6^-methylguanine methyltransferase) promoter methylation and tumor mutational burden (TMB) were associated with mismatch repair (MMR) gene alterations, suggesting that evaluation of MMR genes for both biological and therapeutic considerations may be reasonable in GBM [15].

In this study, we aimed to conduct our WES studies using formalin-fixed, paraffin-embedded (FFPE) primary and recurrent GBM pairs from adult patients in the clinical setting, in order to define if this approach has real-life diagnostic value and may reveal information applicable to the care of individual patients. We used deep bioinformatic analyses for an enhanced and reliable identification of oncogenic and likely oncogenic variants in corresponding primary and recurrent GBM sample pairs, while also defining those variants that persist over time. The samples included here were partly subjects of our previous epigenomic studies [16,17,18].

## 2. Results

### 2.1. WES Coverage and Mapping Quality in the GBM-P and GBM-R Cohorts

The average coverage was 163 ± 63 SD, and the median coverage was 139 in the 10 primary (GBM-P) samples. The fraction of exome with at least 30× coverage was 77% and the median insert size was 132 bp. The mean mapping quality was 57.5, and the percentage of aligned mapped reads was 99%. The average coverage was 186 ± 89 SD and the median coverage was 160 in the 10 recurrent (GBM-R) samples. The fraction of exome with at least 30× coverage was 77% and the median insert size was 129 bp. The mean mapping quality was 57.5, and the percentage of aligned mapped reads was 98% (Table S1).

### 2.2. Genomic Variants in the GBM Sample Pairs

First, we determined the sum of variant numbers in affected genes in the 10 GBM sample pairs. We used a 553 gene-containing glioma panel (Table S2) and the COSMIC database filters to focus only on the glioma-associated variants among the other numerous variants detected by NGS. We excluded the benign and likely benign variants. Subsequently, three variant categories were distinguished based on the VarSeq Cancer Classifier algorithm and scoring system: oncogenic (5+ ≥ score), likely oncogenic (3+ ≥ score) and variant of uncertain significance (VUS) (2+ ≤ score). The software analyses were performed as “duos” of GBM-P and GBM-R samples, and variants were simultaneously evaluated and checked as to whether they exclusively appeared in one of the pairs (GBM-P or GBM-R) or in both (GBM-S). This approach resulted in three groups based on the appearance of variants. Variants that exclusively appeared in primary GBM samples, were sorted into the GBM-*p*, while those that exclusively appeared in recurrent GBM samples were sorted into the GBM-R category. The GBM-S category was created for variants shared between the primary and recurrent samples, thereby representing variants persisting over time. The average number of variants in the primary samples was 24, of which 1 was oncogenic, 3 were likely oncogenic and 20 were VUS. In the recurrent samples, we identified an average of 34 variants, of which 2 were oncogenic, 3 were likely oncogenic and 29 were VUS. The average number of variants occurring in both tumors was five, of which one was oncogenic, one was likely oncogenic and three were VUS (Table 1).

### 2.3. Oncogenic and Likely Oncogenic Variants in the 10 GBM Sample Pairs

**Figure 1:**
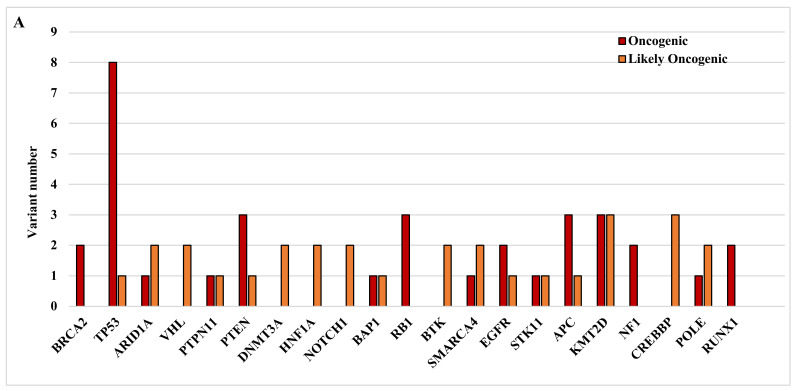

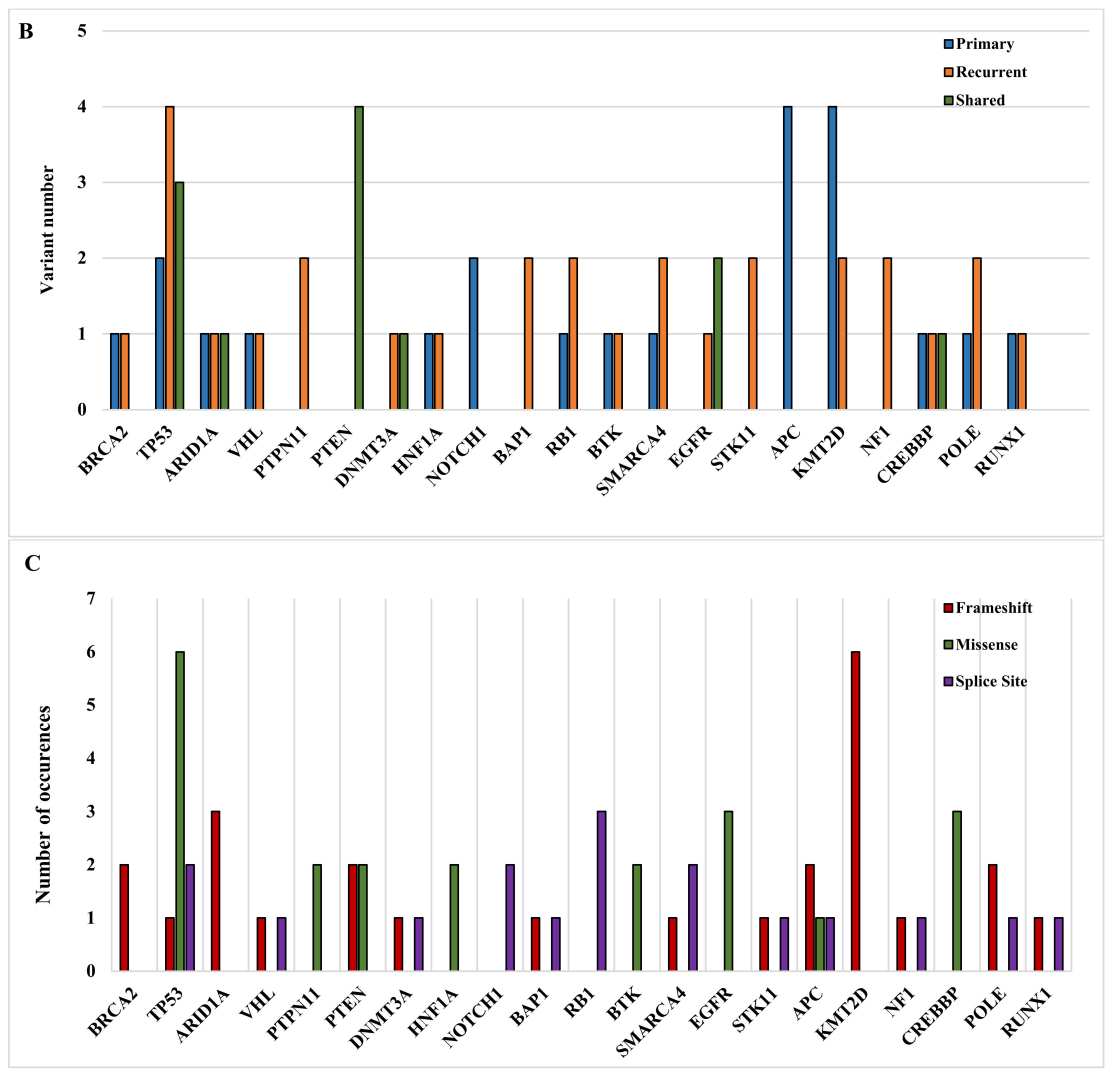
Oncogenic and likely oncogenic variants in our GBM samples. (**A**) depicts oncogenic and likely oncogenic variants in genes as determined by the VarSeq cancer classifier. (**B**) depicts the oncogenic and likely oncogenic variant distributions in the primary, recurrent and shared categories. (**C**) depicts the sequence ontology of detected variants within the affected genes.

In the next step, we analyzed the distribution of oncogenic and likely oncogenic variants in genes that appeared to be affected in more than one patient (Figure 1A–C). Detailed characteristics of all oncogenic and likely oncogenic variants (affected gene, variant allele frequency or VAF, read depth, alternative read count, cancer classifier, COSMIC mutation ID and functional significance) in the three groups (GBM-P, GBM-R, GBM-S) are summarized in Table S3.

Figure 1A–C show the distribution of oncogenic and likely oncogenic variants by gene (A), sample (B) and sequence ontology (C). Not surprisingly, the highest number of oncogenic and likely oncogenic variants were identified in the *TP53* gene, followed by those in the *KMT2 D*, *PTEN*, *APC*, *RB1* and *CREBBP* genes. Of note, *RB1* and *NF1* had only oncogenic variants, just like *BRCA2* and *RUNX1*, while *CREBBP*, *VHL*, *DNMT3 A*, *HNF1 A*, *NOTCH1* and *BTK* carried only likely oncogenic variants (Figure 1A). When we examined the distribution of the gene variants in the primary, recurrent and shared categories, curiously *PTEN* only had variants shared between the primary and recurrent samples. However, there were variants, such as those in *APC* and *NOTCH1*, which were only present in the primary samples or variants, such as those in *PTPN11*, *BAP1*, *STK11* and *NF1*, which were exclusively present in the recurrent samples (Figure 1B). Regarding sequence ontology, the most common variant category was frameshift mutation followed by missense and splice site mutations. *BRCA2*, *ARID1 A* and *KMT2 D* were only affected by frameshift, while *PTPN11*, *HFN1 A*, *BTK, EGFR* and *CREBBP* only by missense and *RB1* and *NOTCH1* only by splice site mutations (Figure 1C).

### 2.4. Variants That also Frequently Occur in Tumors Other Than GBM

We selected variants that were found at least in 1 of our 10 GBM sample pairs and were identified in + ≥15 tumor samples in the COSMIC database. Of the nine highlighted variants, eight were oncogenic, while one was likely oncogenic. Six different variants of *TP53* appeared in our samples, with the most prominent variant being the NM_000546.6:c.742 C>T, NP_000537.3:*p*.Arg248 Trp variant. This variant had 1044 entries in the COSMIC database. In addition to *TP53*, *APC*, *ARID1 A* and *RUNX1* gene variants also frequently appeared in tumors other than glioma in the COSMIC database (Table 2).

### 2.5. Potential Therapeutic Targets Detected in the 10 GBM Sample Pairs

In GBM, currently no approved genetic variant-based therapy is available. We investigated the genes with recurrent oncogenic and likely oncogenic variants in the 10 GBM sample pairs at three levels. First, we checked if there is an available clinical study in any cancer for the identified recurrent genetic variants. Only the *TP53* (NM_000546.6:c.742 C>T, NP_000537.3:*p*.Arg248 Trp; ClViC ID: EID4879) and the *EGFR* (NM_005228.5:c.787 A>C; NP_005219.2:*p*.Thr263 Pro; ClViC ID: EID4187) genes carried variants that have had recent preclinical studies. A subset of 58 cancer cell lines with the *TP53* Arg248 Trp variant appeared insensitive to the MDM2 inhibitor AMGMDS3, while the *EGFR* Thr263 Pro mutant compared to the wild-type Ba/F3 cell line showed some sensitivity to the tyrosine kinase inhibitor (TKI) erlotinib [19,20]. Both aforementioned gene variants have 10+ > COSMIC entries. In our study, the *TP53* variant appeared in the second and fifth, while the *EGFR* variant appeared in the sixth and ninth GBM pairs. The *CREBBP* gene also had a variant (NM_004380.3:c.4394 G>A, NP_004371.2:*p*.Gly1465 Glu) that appeared in more than one sample, namely the sixth, eighth and tenth GBM pairs. However, this latter variant had no targeting drug trial in any cancer (Table S4).

Second, we investigated if there were clinical trials targeting genetic variants in GBM, which were not present in our samples. Only studies targeting variants in the *POLE* (ClViC ID: EID1861) and *RB1* (ClViC ID: EID1595) genes were found. In a GBM case study, a patient with the NM_006231.4:c.1270 C>G; NP_006222.2:*p*.Leu424 Val *POLE* variant was treated with pembrolizumab, an immune checkpoint inhibitor, which resulted in a radiographic response in the intracranial lesion. In a study on GBM cell lines, samples that carried the intact *RB1* gene showed response to palbociclib, a selective inhibitor of CDK4 and CDK6 cyclin-dependent kinases, while samples with homozygous deletions or mutations causing loss of the Rb protein had no palbociclib response [21,22].

Third, for a more comprehensive approach, we considered what the Association for Molecular Pathology (AMP) guidelines [23] suggest for the evaluation of evidence concerning the clinical impact of variants. Successful targeting of certain gene variants falling into Tier IA (FDA-approved therapy available or included in profession guidelines as biomarker) and Tier IB categories (well-powered studies with consensus from field experts shows clinical significance), according to this guideline, may be classified as Tier IIC (FDA-approved therapies in other tumors or investigational therapies available; multiple small studies with consensus) or Tier IID category (preclinical trial or a few case reports without consensus) in gliomas. This consideration would significantly broaden the potential selection of clinical study targets from specific oncogenic or likely oncogenic variants detected in GBM to genes and variants successfully targeted in other tumor types. However, the overview of such extensive potential but not yet established targets is beyond the scope of our study.

### 2.6. Clonality and Tumor Mutation Rate (TMR) in the 10 GBM Sample Pairs

We assessed tumor heterogeneity by clustering variants based on VAF to estimate univariate density and cluster classification (Figure S1). Tumor heterogeneity is inferred by clustering VAFs (using mclust) in the primary and recurrent sample pairs. The MATH score is a simple quantitative measure of intra-tumor heterogeneity, calculated from the width of the VAF distribution. Higher MATH scores are found to be associated with higher clonal heterogeneity of samples. Based on this approach, clonal heterogeneity is slightly increasing in five and decreasing in another five of our sample pairs over time. Table 3 summarizes the results of the calculated tumor mutation rate per megabase for all samples including the total number of mutations, the number of mutations per megabase and the log10-transformed number of mutations per megabase. With the exception of sample pair 7, increases in TMR indicators can be seen in each sample pair. The TMR values used in this study represent an arbitrary measure to reflect changes in the mutation rates when primary and recurrent GBM pairs are compared and should not be confused with the widely used tumor mutation burden (TMB) (see details in the Materials and Methods section):

## 3. Discussion

The widespread use of NGS in studying large numbers of tumor samples in consortial research settings has identified the main molecular drivers of GBM occurrence and recurrence [3,4]. Due to great inter-tumor heterogeneity, determination of the individual molecular profiles may also be necessary. Therefore, we assessed what clinical utility in diagnostics and treatment approaches WES could provide. If we determine the top altered genes and their variants with oncogenic or likely oncogenic significance, can we infer druggable targets with direct clinical benefit for GBM in general and for a given patient in particular? Also, can we gain reliable information using FFPE GBM specimens for NGS?

Regarding technical aspects of NGS, studies have previously shown that quality and number of SNV calls in FFPE samples can be similar to those in freshly-frozen tissue and blood samples [24,25], and the Mutect2 program could detect a similar number of SNVs in FFPE and fresh frozen samples [25,26]. In accordance, we had a relatively high sequencing coverage in both GBM-P (163x ± 63 SD) and GBM-R (186x ± 69 SD) with acceptable DNA quality from our FFPE samples (Table S1). In addition to the recommended high mean coverage, other factors such as application of Mutect2 hard filters, the variant allele frequency (VAF) percentage or alternative read count cutoff and panels of altered genes obtained from tumor databases can greatly influence the variant yield and its reliability [12].

To extract reliable genomic information, we built a stringent filtering pipeline to keep the number of artifacts and false positive variants as low as possible. We used the Mutect2 program for SNV calls, created a filtering pipeline with the exclusion of strand bias and fragment-flagged variants, VAF ≥ 15% and a minimum alternative allele count of ≥20. In addition, we applied the COSMIC filter card, identified oncogenic and likely oncogenic variants based on the new VarSeq Cancer Classifier criteria system (2023.11.15, version 1.0) and implemented a custom-made glioma gene panel based on published data [27,28,29,30,31,32,33] (Table S2). This strict pipeline allowed us to extract clinically important and true genetic variants that presumably contribute to the formation, progression and recurrence of GBM.

In our cohort, the *TP53* gene carried the most variants that appeared in more than half of the GBM pairs (Table S3). In addition, we detected one of the most frequent *TP53* oncogenic variants (indicated in the COSMIC database) in two of our samples, which are currently being targeted in a preclinical study. In this trial, nevertheless, the *TP53* Arg248 Trp variant appeared insensitive to the MDM2 inhibitor AMGMDS3 in a subset of 58 cancer lines (ClViC ID: EID4879) [19]. *TP53* is one of the most commonly mutated tumor suppressor and oncodriver gene in tumors, including GBM. Its mutations disrupt the p53 pathway and induce oncogenic transformation [1,5,7,34,35]. Therefore, clinical monitoring and potential targeting of *TP53* variants may be important elements of personalized medicine in the future.

Following *TP53,* the second-highest numbers of variants were found in the *KMT2 D* gene, which is a histone methyltransferase that modifies histone 3 at lysine 4 (H3 K4) by mono-methylation (H3 K4 me1) [36]. *KMT2 D* is frequently mutated in tumors, including GBM [37], but its normal expression is positively correlated with immune cell infiltration and negatively with tumor mutation burden. Dhar and Lee [38] described that most of the mutations found in *KMT2 D* are missense and truncating mutations, in concordance with our findings (Figure 1C) [38]. Malfunction of the gene product may lead to decreased protein expression, which can contribute to epigenetic changes and affect the mitochondrial metabolic profile [39]. These changes may facilitate tumor development and progression; therefore, *KMT2 D* may be a biomarker and a potential therapeutic target.

The third-highest numbers of variants were found in *PTEN* and *APC*, followed by *SMARCA4*, *CREBBP*, *RB1*, *EGFR*, *ARID1 A* and *POLE*. Similar to *TP53*, *PTEN* is also a known tumor suppressor and oncodriver gene that in a mutated form may contribute to tumor progression and recurrence [40]. In our cohort, the variants in this gene appeared exclusively in shared form (both the primary and recurrent samples) in almost half of the GBM pairs, and were mostly missense. While generally loss of function is the most common form of *PTEN* alterations, missense mutations can also disrupt its tumor-suppressing role and enhance malignant transformation [40]. The *PTEN* wild type and mutation status standalone as well as in combination with other molecular signatures can influence tumor survival time [40], suggesting this gene’s inclusion in clinical tumor panels to serve as a prognostic biomarker.

The *APC* gene contained mostly oncogenic variants, while the *SMARCA4* and *CREBBP* genes most likely contained oncogenic variants. Mutations in *CREBBP* can promote cancer development and progression [41] and act as a tumor suppressor involved in DNA repair mechanisms (i.e., histone or p53 acetylation) [42]. In addition, the gene is part of the WNT pathway modulating glioma stem cell maintenance, differentiation and proliferation [18]. In contrast to the findings by Ellis et al. [43], where three *CREBBP* variants were VUS (one of them also in shared form) [43], our investigation revealed only oncogenic variants of solely missense types. Furthermore, variants of *CREBBP* appeared in all three groups (*p*,R,S), while one of the variants also appeared in three independent GBM pairs. In contrast to *CREBBP*, *APC* variants only appeared in GBM-*p*, while *SMARCA4* variants were predominantly found in GBM-R samples. Altogether, the above results suggest that these genetic variants take part in the initiation and progression of GBM: *APC* in early tumor development and *SMARCA4* in recurrence, while *CREBBP* in both stages. Similar to the *CREBBP* gene products, APC is also a member of the WNT pathway, while *SMARCA4* can interact with elements of the WNT pathway, including *CREBBP* [44]. Altogether, variants in the above-mentioned genes may converge in an oncogenic pathway offering simultaneous targets in GBM.

Various mutations in *EGFR*, a tyrosine kinase receptor gene, are prominent drivers in the development and progression of several cancers, including GBM. In our study, oncogenic and likely oncogenic variants in this gene appeared in all three investigation categories (GBM-*p*, GBM-R and GBM-S). One of the *EGFR* variants (Thr263 Pro) recurred in two GBM sample pairs, and is currently under testing in a cell line-based preclinical trial (ClViC ID: EID4187) [21]. In consensus with previous data, all *EGFR* mutations in our cohort were missense variants, suggesting that this mutation type is the most common somatic *EGFR* SNV alteration [35,45]. Following the great clinical success in other cancers, experimental targeting of EGFR in gliomas has also been intensively studied in both preclinical and clinical settings, as explained later. It is also important to note that WES is not the ideal approach to detect the most common EGFR alterations, CNVs such as the EGFRvIII (exon 2–exon 7 deletion) variant or gene amplification in FFPE GBM samples. Due to the confounding effect of DNA fragmentation, we did not determine copy number alterations genome-wide in our specimens. We mention here for completeness the *EGFR* CNVs, along with the gene’s extensive therapeutic targeting in preclinical and clinical studies, though only with limited success thus far [46].

The mutation rate of the cell cycle and apoptosis regulator *RB1* [47] was lower in our primary samples than those indicated in the TCGA [1]. Just as in the case of previously detailed genes, *RB1* mutations also have tumor-promoting effects and disrupt normal cell biology in many cancers, including GBM [47]. Our findings are in agreement with those by Barthel et al. [10], stating that driver gene mutations in *EGFR*, *NF1* and *RB1* usually persist in the recurrent samples [10].

A review paper by the GLASS consortium [7] discerned various patterns of mutational profiles in primary and recurrent glioma samples. In one study, new mutations developed in the recurrent samples as a result of temozolomide treatment, while in another study, some recurrent tumors carried the same *TP53* variants as the primary samples, while yet another study showed that variants in the recurrent tumors were driven by branched subclonal evolution and did not appear in the primary sample. When linear evolution was observed, the initial and recurrent tumor profiles matched [7]. Of note, from all the analyzed genes in the 10 GBM pairs, only the seventh patient lacked any oncogenic or likely oncogenic variants in the recurrent sample, or an increase in TMR (Table 1 and Table 3). Moreover, only this patient did not receive temozolomide that is known to alter the molecular evolution pattern, including occurrence of hypermutation in recurrent GBM [48]. Eskilsson and Verhaak [49] observed that some recurrent tumors deviated from their primary mutated gene patterns due to subclonal evolution and tumor heterogeneity, while others showed a linear evolution [49]. While our limited data from a small FFPE sample cohort do not allow us to infer a general clonal evolution pattern, they suggest both decreasing and increasing clonal heterogeneity over time (Figure S1). Variants that exclusively appeared in the primary or recurrent samples (i.e., *APC*, *NOTCH1* in GBM-P and *PTPN11*, *BAP1*, *STK11* and *NF1* in GBM-R) probably are signs of branching clonal evolution patterns influenced, at least in part, by treatment effects [14], while the detection of shared variants aligned more with the linear evolution pattern (i.e., *PTEN*, *EGFR*, *TP53*).

The above data indicate that WES is suitable for the molecular characterization of individual clinical samples and is capable of identifying one or more drivers of tumor development. As these variants align in critical pathways of tumorigenesis, simultaneous targeting of multiple pathway elements might be considered for treatment development. Nonetheless, in this study we asked the question as to whether identification of oncogenic drivers in the clinical setting presently has direct utility in the treatment of a given patient with GBM.

Several approaches have been developed or are under development to block selected oncogenic drivers. The short list includes small molecular inhibitors, monoclonal antibodies (mAbs), specific vaccines and engineered immune cells. Regulatory RNAs (microRNA, siRNA, lncRNA etc.) and mRNA vaccines are relatively new and potent tools in tumor therapy because of the flexibility and adaptability of the techniques [50]. The mutation rate of *TP53* is relatively high in GBM [8,11]; therefore, therapeutic targeting of this gene product is constantly under development [51]. Members of the tyrosine kinase receptor (RTK), PI3 K and RAS signaling pathways, such as EGFR, MEK and NF1, can be modulated by small molecular inhibitors and antibodies [50]. EGFR is one of the main driver molecules in GBM and has been targeted by CAR-T cells, vaccines (rindopepimut), small inhibitors, mAbs (cetuximab) and antibody–drug conjugates (Depatux-M, ABBV–221) in phase I, II or III trials. Though some approaches had been quite promising in the early phases, at the end, none of the studies have thus far yielded successful treatment in GBM, in contrast to other tumors [50]. EGFR RTK inhibitors have been particularly successful in non-small cell lung cancer, but appeared ineffective when tested in GBM. One could postulate that the reason might be that the compounds used in lung cancer inhibit the intracellular (tyrosine kinase) domain of the receptor where the mutations are also located, while the extracellular domain is mostly mutated in GBM. However, this mutated (e.g., EGFRvIII) receptor in GBM is also constitutively active through the phosphorylation of the down-stream signaling molecules by the intracellular tyrosine kinase domain. Therefore, the lack of efficacy of tyrosine kinase inhibitors in GBM may be related to reasons other than the different sites of mutations. Similarly, other drugs successfully targeting certain genes or variants in a variety of tumors may not be equally effective in GBM for many reasons related to the blood–brain barrier, the tumor microenvironment, molecular and biochemical properties of different tumors and host factors outside of the tumors. Therefore, until a clinical trial establishes the clinical benefit of targeting any given mutated gene or its oncogenic variant in GBM, inferring success from the promising outcome of trials in other cancers cannot be supported by the available data. Altogether, we may conclude that determination of the mutational profiles and driver genes in clinical GBM specimens is feasible by WES. However, presently it should be restricted to carefully selected patients for whom the standard medication has been exhausted and some benefit from a targeted therapy may be expected based on individual tumor characteristics.

Our study has some strength and weakness. We defined characteristic genomic changes and dynamics in sequential GBM sample pairs in the interface of research and translation. We used deep bioinformatics analyses to extract as many and as reliable variants relevant to gliomagenesis as possible. Our data support the utility of NGS in the diagnostic work up, even of individual GBM samples, but our analyses show as yet limited real-life therapeutic applicability. The use of DNA from FFPE samples and the applied bioinformatic analyses also involve some interpretive difficulties. The DNA modification by formalin greatly contributed to high numbers of false positive artefacts, but we made all efforts to bioinformatically minimize this effect by filtering out strand bias and fragment-flagged variants. As we studied DNA from a small cohort of FFPE samples, we used the COSMIC database and a glioma gene panel to identify the most relevant variants in GBM. This approach obviously prevented the discovery of new pathogenic variants, and made obligatory (if performed from the data of our small cohort) identifications of already known affected molecular pathways; however, our research did not aim for scientific discovery, rather to establish clinical utility of the variant detection. Overall, our analyses provided new information regarding molecular gliomagenesis and diagnostics, and support a careful as well as selective clinical applicability of this approach in clinical settings.

## 4. Materials and Methods

### 4.1. Subjects of the Study

Surgically removed FFPE GBM specimens were obtained between 1999 and 2017 at the Department of Pathology, University of Pecs (UP). Blocks left over from clinicopathological work up were used for the present molecular analyses after receiving approval (number: 7517 PTE 2018) from the Regional Clinical Research Ethics Committee of UP. After quality assessment, 10 pairs of immunohistochemistry screened, isocitrate dehydrogenase (IDH)−1 R132 H mutation negative initial (GBM-P) and recurrent (GBM-R) tumor blocks were included from patients with late onset disease [age range: 39–66 years] (Table 4).

### 4.2. Sample Characteristics

The diagnosis of primary (de novo) GBM was established based on standard clinical and histopathological criteria at the time when the study was initiated [3] and later upgraded according to the 2021 WHO CNS5 criteria [4]. Primary tumor specimens were taken before and recurrent specimens after chemoradiation.

**Figure 2:**
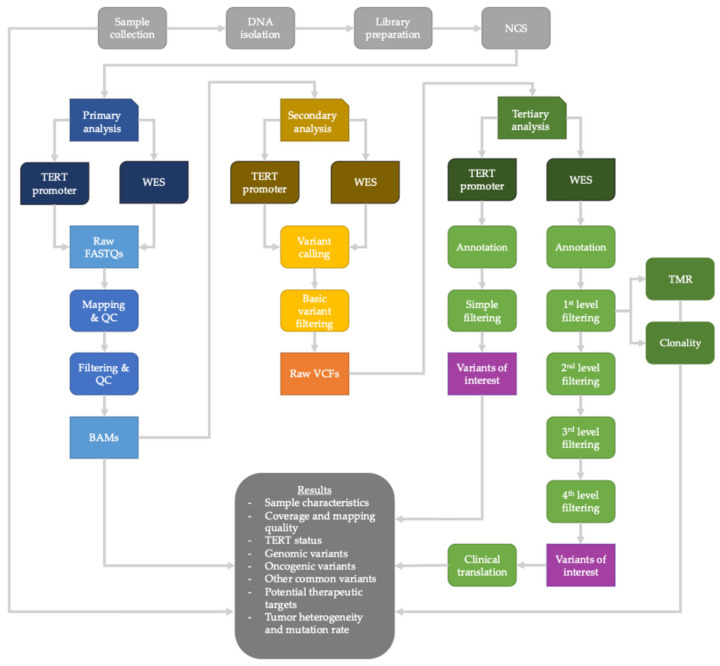
Workflow of laboratory preparations and bioinformatic analyses.

In addition to histological features of GBM (mitotic index, microvascular proliferation, necrosis, atypia), molecular characteristics inferred from WES also supported the WHO CNS5-based diagnosis of GBM [4]. All GBM-P and -R specimens were *IDH1/2* wild type by WES. Of the 10 GBM-P samples, 8 had *TERT* promoter mutation and 6 showed *EGFR* amplification. Of the 10 GBM-R samples, 9 had *TERT* promoter mutation and 7 had *EGFR* amplification. We identified homozygous *CKDN2 A* deletions in two and two of the ten (20%) GBM-P and GBM-R samples, respectively. In one case, where the primary tumor had a homozygous deletion, the corresponding recurrent sample contained a heterozygous deletion, while in another sample pair, the reverse of these changes was noted. Heterozygous deletions of the *CDKN2 A* gene were noted in one GBM-P and three additional GBM-R samples (Table S5). All samples satisfied the latest WHO CNS5 criteria for GBM diagnosis considering the histopathological and molecular characteristics [4] (Figure 2).

### 4.3. Sample Preparation and Quality Check

DNA was isolated from FFPE tissue sections received from the Department of Pathology of the University of Pecs (UP). The samples were deparaffinized with xylene and, after washing with alcohol, the QIAamp DNA FFPE tissue kit (Qiagen^®^, Hilden, Germany) was used for the isolation of DNA. In the final step, DNA was eluted from the columns in 50 µL of buffer and stored at −20 °C until further use. The concentration of the eluate was determined using the Qubit™ 1 X dsDNA HS assay kit on a Qubit 4 fluorimeter (Invitrogen, Carlsbad, CA, USA). Fragment analysis of the eluted DNA was performed using the Agilent Genomic DNA ScreenTape assay kit on an Agilent 4200 TapeStation system (Agilent Technologies, Santa Clara, CA, USA). In the primary samples, the average rate was 50.34 ± 13.91% SD between 200 and 2000 bp fragments, while the average rate was 36.76 ± 8.75% between 2000 and 60,000 bp fragments. In the recurrent samples, the average rate was 58.13 ± 11.75% SD between 200 and 2000 bp fragments, while the average rate was 29.61 ± 9.12% between 2000 and 60,000 bp fragments (Figure 2, Table S6).

### 4.4. TERT Promoter Sequencing

*TERT* promoter PCR products were sequenced using NGS to determine the mutational status of two well-known hotspots (C228 T and C250 T). The hotspot-containing region was amplified using GoTaq^®^ Long PCR Master Mix (Promega, Madison, WI, USA) and primers based on the paper by Hafezi et al. [52]. Amplicon libraries were prepared using the NEBNext Ultra II DNA library prep kit (NEB, Ipswitch, MA, USA) and sequencing was performed on a NovaSeq 6000 instrument (Illumina, San Diego, CA, USA) (Figure 2).

### 4.5. Library Preparation for WES

Library preparation and exome capture were performed using Sure Select Human All Exon V7 (Agilent Technologies, Santa Clara, CA, USA). Briefly, 50 ng of DNA was enzymatically fragmented, end prepped, adaptor ligated and amplified. Hybridization was performed using Human All Exon V7 probes (Agilent Technologies, Santa Clara, CA, USA). Libraries were qualitatively and quantitatively assessed using TapeStation 4200 (Agilent Technologies, Santa Clara, CA, USA) and Qubit 3.0 (Invitrogen, Carlsbad, CA, USA) and were sequenced on a NovaSeq 6000 machine (Illumina Inc. San Diego, CA, USA) for 2 × 150 paired-end reads (Figure 2).

### 4.6. Bioinformatics

Raw FASTQ file generation, including basecall and demultiplexing, was carried out using bcl2 fastq (v2.20.0.422) [53] on a local HPC cluster. First, raw data quality was checked using FastQC (v0.11.9) [54]. Based on the results, datasets were filtered and adapters and low-quality bases/reads were removed with fastp (v0.21.0) [55]. Clean, high-quality sequences were aligned to the human reference genome (GRCh37), applying the bwa mem algorithm (v0.7.17) [56]. Default parameters were used for the chromosomes. The SAM/BAM file modifications (e.g., sorting, adding read group information, indexing) were carried out using various Picard Tools (v2.23.3) subcommands [57]. Before variant calling, base quality scores were recalibrated by the GATK BSQR module (v4.1.4.1) [58]. Somatic SNVs and short insertions and deletions (INDELs) were identified with the GATK Mutect2 algorithm [59]. Finally, raw variants were pre-filtered using the GATK FilterMutectCalls module [59] (Figure 2).

### 4.7. Filtering Pipeline for Variant Selection

VarSeq software version 2.5.0 (Golden Helix, Inc., Bozeman, MT, USA) was used for annotation and variant filtering (SNVs and INDELs). The GBM pairs were uploaded as duos.

First level of filtering: Hard filter fragment and strand bias-flagged variants were filtered out using Mutect2. Read depth was set to equal or higher than 50, and VAF was set to equal or higher than 15%. Variants with alternative (Alt) read counts lower than 20 were filtered out. Variants were removed with equal or higher than 2% allele frequencies (in 1 kG Phase3–Variants 5 a with genotype counts), equal or higher than 2% alternative allele frequencies (in gnomAD Exome Variant frequencies 2.1.1) and equal or higher than 5% minor allele frequencies (in NHLBI ESP6500 SI-V2-SSA137 Exome Variant Frequencies 0.0.30). Variants with dbSNP (dbSNP Common 155, NCBI) entries were also filtered out.

Second level of filtering: Variants flagged as initiator codon, intragenic, intron and synonymous were filtered out (Sequence Ontology, RefSeq Genes 105.20220307, NCBI). Functional interpretation of the variants was provided by dbNSFP in silico prediction tools (dbNSFP Functional Prediction Voting) (i.e., SIFT, PolyPhen2 HVAR, MutationTaster, MutationAssessor, FATHMM and FATHMM MKL), and variants that were predicted to be damaging, at least in one, were filtered out. Clinically nonsignificant variants (Benign, Likely Benign, VUS/Weak Benign) were filtered out based on the VarSeq Clinical Classification criteria system. Variants with loss of function (LoF), missense mutation, Other or Unknown predicted effect (RefSeq Genes 105.20220307, NCBI) and with at least one record in the COSMIC database (Cosmic Mutations 96, GHI) were retained. As a last step, we used the Mendel error filter card to extract variants with Shared, Unshared and de Novo Allele tags. The detectability of the variants depended on many factors including DNA fragmentation caused by the FFPE conservation technique, and the strict filter pipeline intended for avoiding artifacts. As a result of the latter, variants of decreased quality were filtered out in the primary or recurrent samples. However, in order to better capture disease-relevant variants, Varseq analyses were performed with “duos” of GBM-P and GBM-R variants, meaning that variants that passed the filters in one sample were specifically searched for in the corresponding sample pair. Using this approach, several variants were identified to be shared between the sample pairs (designated as GBM-S).

Third level of filtering: Next, variants were filtered using a custom-made glioma gene panel based on prior scientific publications [27,28,29,30,31,32,33]. Fusion, Oncogen and Tumor Suppressor variants were retained based on the COSMIC Cancer Gene Consensus 96, GHI database.

Fourth level of filtering: We manually curated and interpreted the remaining variants with VarSeq, VSClinical AMP (Figure 2, Table 5).

### 4.8. Identification of Oncogenic and Likely Oncogenic Variants in GBM-p, GBM-R and GBM-S

GBM-P and GBM-R NGS data obtained at filter level 2 were loaded into VarSeq as related duo samples. By this approach, we were able to search for and evaluate variants that appeared exclusively in the GBM-P and GBM-R categories, or appeared in both designated as shared category (GBM-S) (in other words, variants that persisted over time from primary to recurrent stages).

### 4.9. Tumor Mutation Rate (TMR)

TMR values were estimated by defining all variants (with at least 20× depth and after level 1 filtering in Table 5) per megabase. The capture size was set up to 42 MB. In addition to the total numbers of mutations used in the calculation, the numbers of mutations per megabase and the log10-transformed numbers of mutations per megabase were defined. The determination of TMR in this study differs from that generally used for the determination of tumor mutation burden (TMB). Due to the low number of samples and data points, the TMB values (typically calculated from oncogenic, likely oncogenic and VUS/weak oncogenic variants) would be too low in this study and would not be comparable with those calculated from data of large research cohorts. The purpose of TMR generated from the variants defined above is to reveal the changes in mutation rates from the primary to the recurrent stages of disease.

### 4.10. Tumor Heterogeneity

Variant allele frequency (VAF) values of all variants after level 1 filtering in Table 5 were used to calculate tumor heterogeneity by applying the default parametric finite mixture model (mclust) for clustering [60]. To measure intra-tumor heterogeneity, Mutant-Allele Tumor Heterogeneity (MATH) scores were calculated as well. The MATH score is a non-biased, quantitative method to assess intra-tumor genetic heterogeneity based on NGS-generated data. It is calculated as the ratio of the width to the center of distribution of mutant allele fractions among tumor-specific mutated loci [60,61]. MATH values of our GBM samples were calculated from the median absolute deviation (MAD) and the median of its mutant allele fractions at tumor-specific mutated loci using the built-in math.score function (https://rdrr.io/bioc/maftools/src/R/mathScore.R, accessed on 4 July 2024) from the maftools package [62], based on the following formula: MATH = 100 * MAD/median. Higher MATH scores are associated with higher heterogeneity of samples.

## 5. Conclusions

Our analyses reflect that the WES method is suitable, but with caution, for studying DNA from FFPE glioma specimens, as it is capable of providing valuable information regarding somatic oncogenic variants in smaller cohorts and individual samples in the clinical setting. In addition to supporting the latest WHO classifications of gliomas, this NGS-based method also allows us to monitor molecular tumor changes and identify the main molecular drivers at onset and at recurrence. The gained information can support patient selection for preclinical and clinical studies, or allow complementing standard treatment protocols in selected patients. Along with the published data, our results suggest that combined and personalized therapeutic interventions may be needed to successfully treat GBM. Therefore, clinical application of comprehensive (WES- or panel-based) molecular diagnostic approaches will likely gain wider roles in testing individual clinical GBM samples in the near future.

## Figures and Tables

**Table 1:** Variant numbers with different oncogenicity category across the 10 GBM pairs. GBM-P: Primary sample variants, GBM-R: Recurrent sample variants, GBM-S: Shared variants that appeared in both GBM-P and -R samples. O = Oncogenic, LO = Likely oncogenic, VUS = Variant of uncertain significance.

GBM Pairs	GBM-P				GBM-R				GBM-S			
	SUM	O	LO	VUS	SUM	O	LO	VUS	SUM	O	LO	VUS
1	29	3	3	23	27	0	2	25	9	2	1	6
2	12	0	3	9	26	1	0	25	4	2	0	2
3	13	0	5	8	55	3	5	47	3	0	1	2
4	12	1	0	11	24	3	4	17	2	0	0	2
5	61	1	5	55	61	5	2	54	5	1	0	4
6	13	1	1	11	35	3	4	28	7	1	2	4
7	36	2	5	29	17	0	0	17	1	0	0	1
8	34	3	2	29	34	2	8	24	6	3	0	3
9	10	0	1	9	14	1	0	13	4	1	1	2
10	16	1	1	14	41	3	2	36	6	1	1	4
Mean	24	1	3	20	34	2	3	29	5	1	1	3

**Table 2:** Variants in GBM also frequently detected in other tumors. The table shows variants that are frequent in the COSMIC database. Missense variant = M, Frameshift = F, Splice variant = SV, O = Oncogenic, LO = Likely oncogenic.

Variant Info		Gene	Cancer Classifier					COSMIC Mutations 96	
Chr:Pos	Ref/Alt	Gene Name	HGVS cDot	HGVS pDot	Seq. Ont.	Score	Class.	Mutation ID	Count
17:7578205	C/T	TP53	NM_000546.6:c.644 G>A	NP_000537.3:*p*. Ser215 Asn	M	9	O	COSM44093	29
17:7578262	C/G	TP53	NM_000546.6:c.587 G>C	NP_000537.3:*p*. Arg196 Pro	M	9	O	COSM43814	36
17:7577539	G/A	TP53	NM_000546.6:c.742 C>T	NP_000537.3:*p*. Arg248 Trp	M	9	O	COSM10656	1044
17:7578291	T/A	TP53	NM_000546.6:c.560–2 A>T	*p*.?	SV	4	LO	COSM45026	15
17:7578550	G/T	TP53	NM_000546.6:c.380 C>A	NP_000537.3:*p*. Ser127 Tyr	M	8	O	COSM43970	37
21:36171607	G/A	RUNX1	NM_001754.5:c.958 C>T	NP_001745.2:*p*. Arg320 Ter	F	7	O	COSM41699	21
1:27087503	C/T	ARID1 A	NM_006015.6:c.2077 C>T	NP_006006.3:*p*. Arg693 Ter	F	6	O	COSM184236	37
5:112164616	C/T	APC	NM_000038.6:c.1690 C>T	NP_000029.2:*p*. Arg564 Ter	F	9	O	COSM18848	82
17:7577548	C/T	TP53	NM_000546.6:c.733 G>A	NP_000537.3:*p*. Gly245 Ser	M	9	O	COSM6932	670

**Table 3:** Tumor mutation rates in GBM-P and GBM-R samples. The table shows the tumor mutation rates (TMRs) in the 10 GBM pairs. The TMR values were calculated by defining all variants (with at least 20× depth and after level 1 filtering, see details in Section 4.3) per megabase. GBM-P: Primary sample variants, GBM-R: Recurrent sample variants.

Sample	Total Mutations	Total Mutations per Mb	Total Mutations per Mb (log10)
1. GBM-P	15,227	362.55	2.56
1. GBM-R	25,619	609.99	2.79
2. GBM-P	11,898	283.29	2.45
2. GBM-R	16,759	399.02	2.6
3. GBM-P	6281	149.55	2.17
3. GBM-R	39,456	939.43	2.97
4. GBM-P	11,858	282.33	2.45
4. GBM-R	22,414	533.67	2.73
5. GBM-P	35,395	842.74	2.93
5. GBM-R	45,060	1072.86	3.03
6. GBM-P	9111	216.93	2.34
6. GBM-R	20,473	487.45	2.69
7. GBM-P	20,877	497.07	2.70
7. GBM-R	10,648	253.52	2.40
8. GBM-P	17,404	414.38	2.62
8. GBM-R	22,285	530.6	2.72
9. GBM-P	6119	145.69	2.16
9. GBM-R	7341	174.79	2.24
10. GBM-P	11,005	262.02	2.42
10. GBM-R	29,776	708.95	2.85

**Table 4:** Clinical characteristics of patients. The table shows patients’ gender, age of onset of GBM, applied treatment and time to relapse in weeks.

GBM-P Samples	GBM-R Samples	Gender	Age at Onset	Treatment	Time to Relapse (Weeks)
UPL22–003804	UPL22–003816	man	61	Surgery + radio + TMZ	49
UPL22–003805	UPL22–003817	man	39	Surgery + radio + TMZ	40
UPL22–003806	UPL22–003818	man	62	Surgery + radio + TMZ	58
UPL22–003807	UPL22–003819	woman	61	Surgery + radio + TMZ	31
UPL22–003810	UPL22–003822	man	66	Surgery + radio + TMZ	56
UPL22–003811	UPL22–003823	woman	53	Surgery + radio + TMZ	55
UPL22–003812	UPL22–003824	woman	63	Surgery+ irradiation	30
UPL22–003813	UPL22–003825	woman	45	Surgery + radio + TMZ	143
UPL22–003814	UPL22–003826	man	43	Surgery + radio + TMZ	135
UPL22–003815	UPL22–003827	woman	56	Surgery + radio + TMZ	199

**Table 5:** VarSeq filtering pipeline. This table summarizes filter cards, databases and settings applied in four sequential steps when creating our analysis pipeline.

	Filter Cards and Databases	Settings
First level of filtering	GATK Mutect2 hard filters	Fragment and SB variants were filtered out
	Read Depth (DP)	≥50
	Variant Allele Frequency (VAF)	≥15%
	Alternative Read Count	≥20
	Allele Freq (1 kG Phase3)	≥2% or missing
	Alternative Allele Freq	≥2% or missing
	(gnomAD Exome Variant frequencies 2.1.1)	
	All Minor Allele Frequency (NHLBI 0.0.30)	≥5% or missing
	dbSNP Common 155 (NCBI)	False
Second level of filtering	Sequence Ontology (RefSeq Genes 105.20220307, NCBI)	Initiation codon, intragenic and synonymous variants were filtered out
	dbNSFP Functional Prediction Voting	Functional interpretation of variants
	Cancer Classifier	Benign, Likely Benign, VUS/Weak benign and missing variants were filtered out
	Effect (RefSeq Genes 105.20220307, NCBI)	LoF, missense, other and missing variants were retained
	COSMIC (Cosmic Mutations 96, GHI)	True
	VarSeq built-in flag system	Technically hard to detect variant extraction
Third level of filtering	GeneID (Aux Fields RefSeq Genes 105.20220307, NCBI)	553 gene glioma-specific panel
Fourth level of filtering	VSClinical, AMP	Manual variant interpretation

## Data Availability

Raw data are available at the European Nucleotide Archive (ENA) under PRJEB63610 accession.

## Supplementary Materials

The following supporting information can be downloaded at: https://www.mdpi.com/article/10.3390/ijms25147564/s1.
