# Supplementary material for: On the Boundary of Exploratory Genomics and Translation in Sequential Glioblastoma
Source: Int J Mol Sci. 2024 Jul 10;25(14):7564. doi: 10.3390/ijms25147564 (PMC11277311; doi:10.3390/ijms25147564)
Supplement: Supplementary file 1 [file ijms-25-07564-s001.zip › Supplementary figure S1.pdf]

On the Boundary of Exploratory Genomics and Translation in Sequential Glioblastoma

Marton Tompa [1,2,*], Bence Galik [1], Peter Urban [1], Bela Istvan Kajtar [3], Zoltan Kraboth [3], Attila Gyenesei [1], Attila Miseta [4] and Bernadette Kalman [1,2,4,*]

1 Szentagothai Research Center, University of Pecs, 20. Ifjusag Street, 7624 Pecs, Hungary; galik.bence@pte.hu (B.G.); urban.peter@pte.hu (P.U.); gyenesei.attila@pte.hu (A.G.)

2 Department of Molecular Medicine, Markusovszky University Teaching Hospital, 5. Markusovszky Street, 9700 Szombathely, Hungary

3 Department of Pathology, School of Medicine, University of Pecs, 12. Szigeti street, 7624 Pecs, Hungary; kajtar.bela@pte.hu (B.I.K.); kraboth.zoltan@pte.hu (Z.K.)

4 Office of the Dean, School of Medicine, University of Pecs, 20. Ifjusag Street, 7624 Pecs, Hungary; attila.miseta@aok.pte.hu

* Correspondence: tompa.marton@pte.hu (M.T.); bernadette.kalman@pte.hu (B.K.)

A

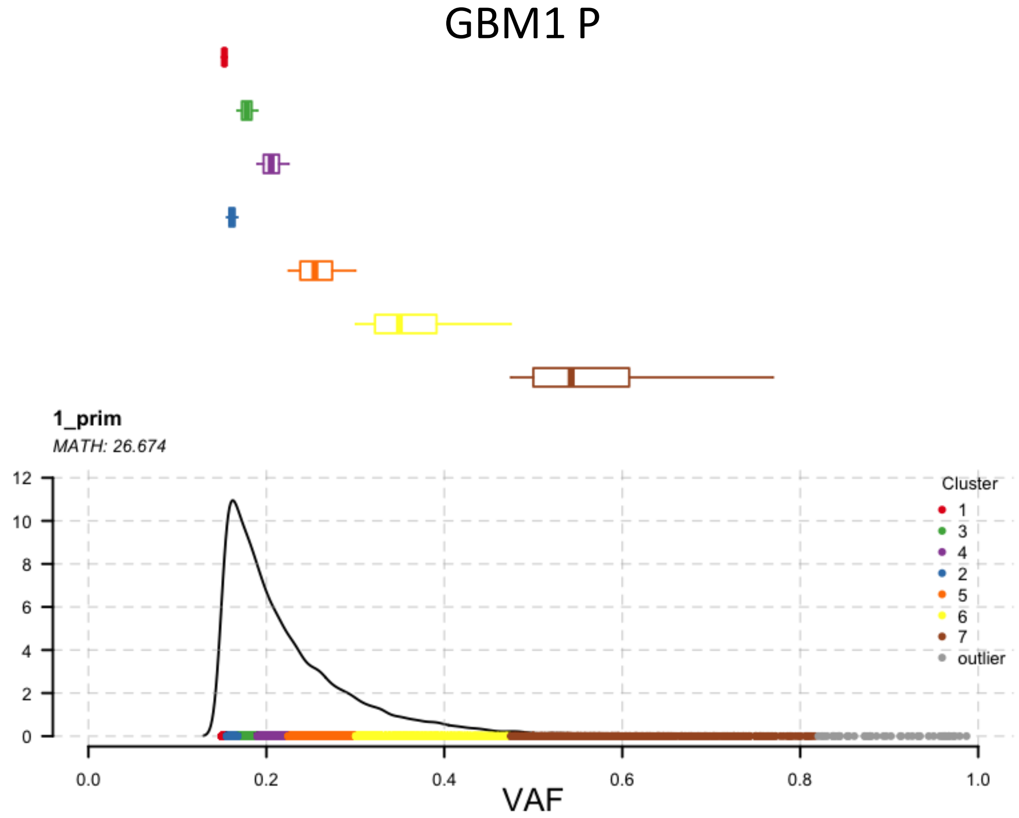

B

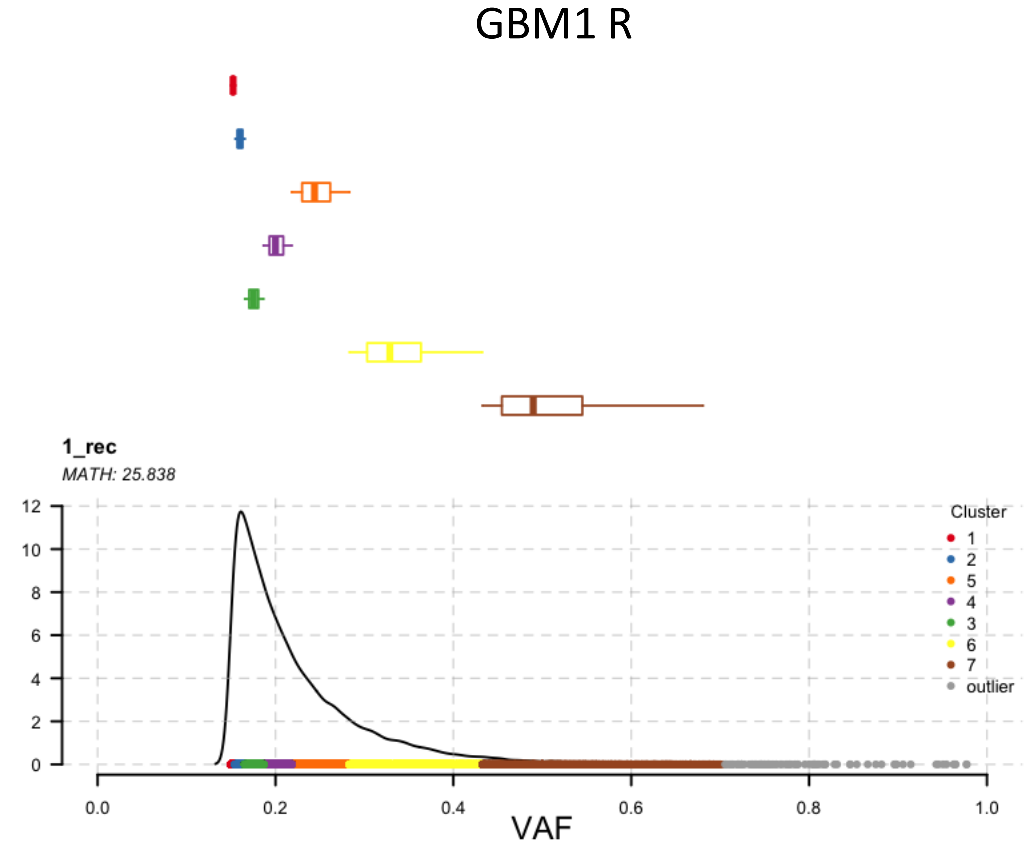

C

cluster	meanVaf	Min	25perc	Median	75perc	Max	No. MUTs	No. Genes
1	0.1527525	0.150000	0.151316	0.1527780	0.154286	0.155556	3071	2087
3	0.1776390	0.167382	0.172131	0.1774190	0.183099	0.188811	8122	4549
4	0.2051084	0.188841	0.196262	0.2040820	0.213592	0.224599	8762	4888
2	0.1612769	0.155629	0.158273	0.1612900	0.164062	0.167364	5370	3330
5	0.2561669	0.224638	0.237113	0.2531650	0.273438	0.299401	8945	5017
6	0.3586537	0.300000	0.320000	0.3486610	0.390244	0.473684	5064	3258
7	0.5665907	0.473988	0.500000	0.5416670	0.605263	0.816327	853	723
outlier	0.8991370	0.820225	0.844311	0.8896655	0.959184	0.986842	54	45

D

cluster	meanVaf	Min	25perc	Median	75perc	Max	No. MUTs	No. Genes
1	0.1522937	0.150000	0.151079	0.152318	0.153465	0.154762	4698	3149
2	0.1599933	0.154812	0.157360	0.160000	0.162602	0.165480	9365	5376
5	0.2454570	0.217778	0.229358	0.243243	0.260870	0.282258	15393	7391
4	0.2003544	0.185484	0.192308	0.200000	0.208333	0.217742	15257	7508
3	0.1750438	0.165517	0.170000	0.174825	0.180124	0.185430	14155	7183
6	0.3353604	0.282353	0.302326	0.327869	0.363636	0.431138	10059	5462
7	0.5061587	0.431373	0.453125	0.488889	0.542857	0.700000	1974	1528
outlier	0.7928590	0.703704	0.735779	0.767857	0.818182	0.977273	99	92

A

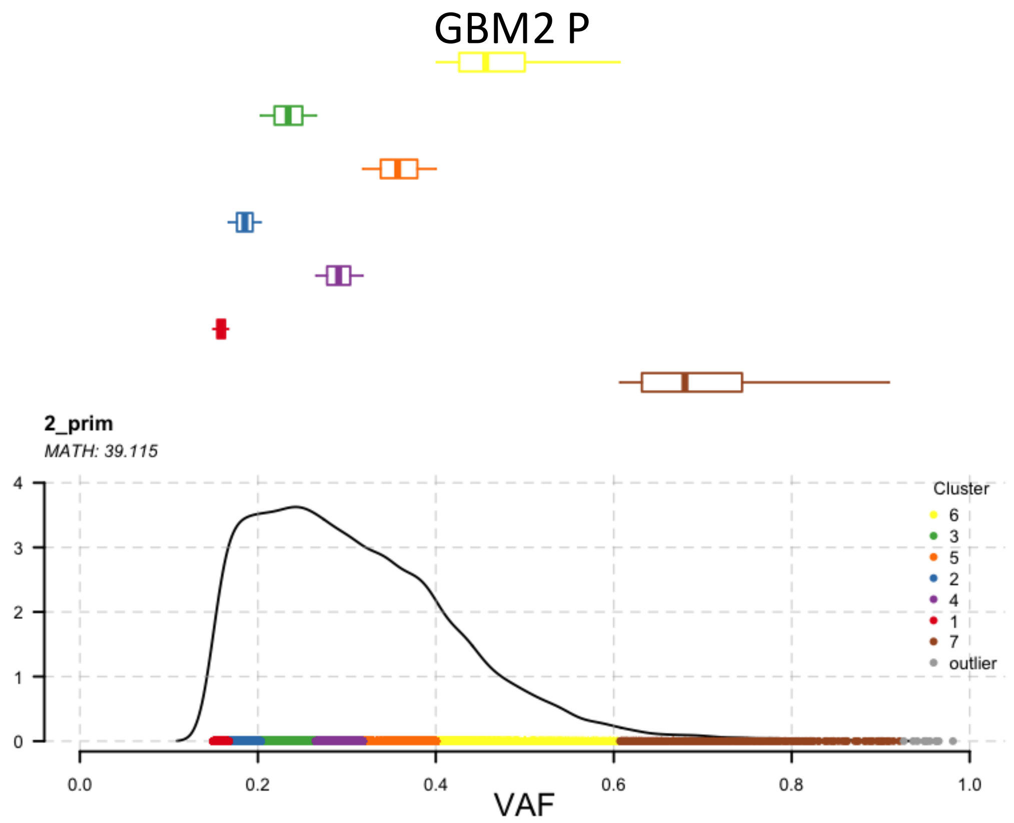

B

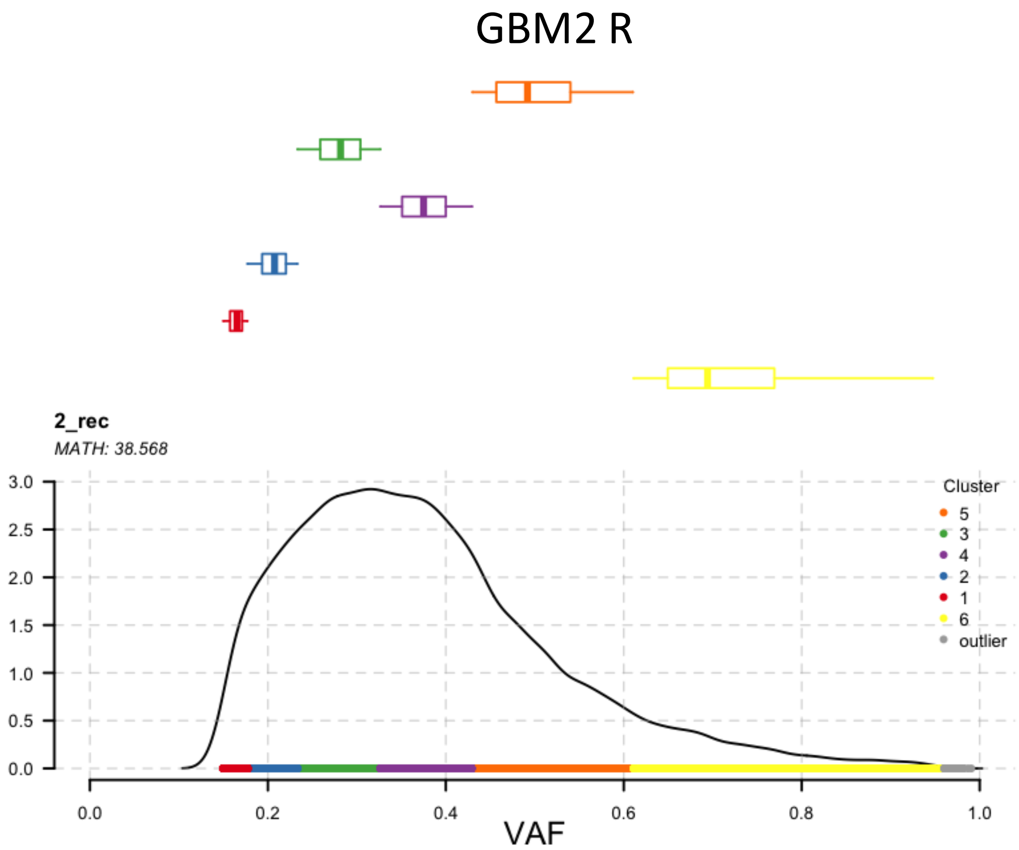

C

cluster	meanVaf	Min	25perc	Median	75perc	Max	No. MUTs	No. Genes
6	0.4654224	0.400000	0.4223180	0.452830	0.5000000	0.604167	4760	2912
3	0.2341324	0.203125	0.2187500	0.234043	0.2500000	0.265487	5694	3190
5	0.3562672	0.318182	0.3375000	0.354839	0.3768120	0.398601	5460	3166
2	0.1851837	0.167116	0.1764710	0.185185	0.1941750	0.203046	3092	1850
4	0.2910166	0.265625	0.2777780	0.290739	0.3037970	0.317757	4362	2623
1	0.1586562	0.150000	0.1542860	0.158730	0.1631210	0.166667	1461	957
7	0.6978599	0.605263	0.6315790	0.680000	0.7441860	0.925926	478	359
outlier	0.9550227	0.936508	0.9495765	0.951613	0.9626135	0.981132	11	11

D

cluster	meanVaf	Min	25perc	Median	75perc	Max	No. MUTs	No. Genes
5	0.4989179	0.428571	0.4545450	0.490566	0.5384620	0.608696	7462	4021
3	0.2810656	0.233333	0.2588240	0.281690	0.3043480	0.327434	8782	4313
4	0.3753272	0.327586	0.3506490	0.375000	0.4000000	0.427632	9178	4656
2	0.2068395	0.177083	0.1935480	0.207547	0.2203390	0.233129	4030	2268
1	0.1643290	0.150000	0.1574800	0.165289	0.1710530	0.176991	1480	911
6	0.7168369	0.609375	0.6470590	0.692308	0.7692310	0.955224	2489	1526
outlier	0.9721537	0.956522	0.9645325	0.972973	0.9799055	0.990566	44	36

A

GBM3 P

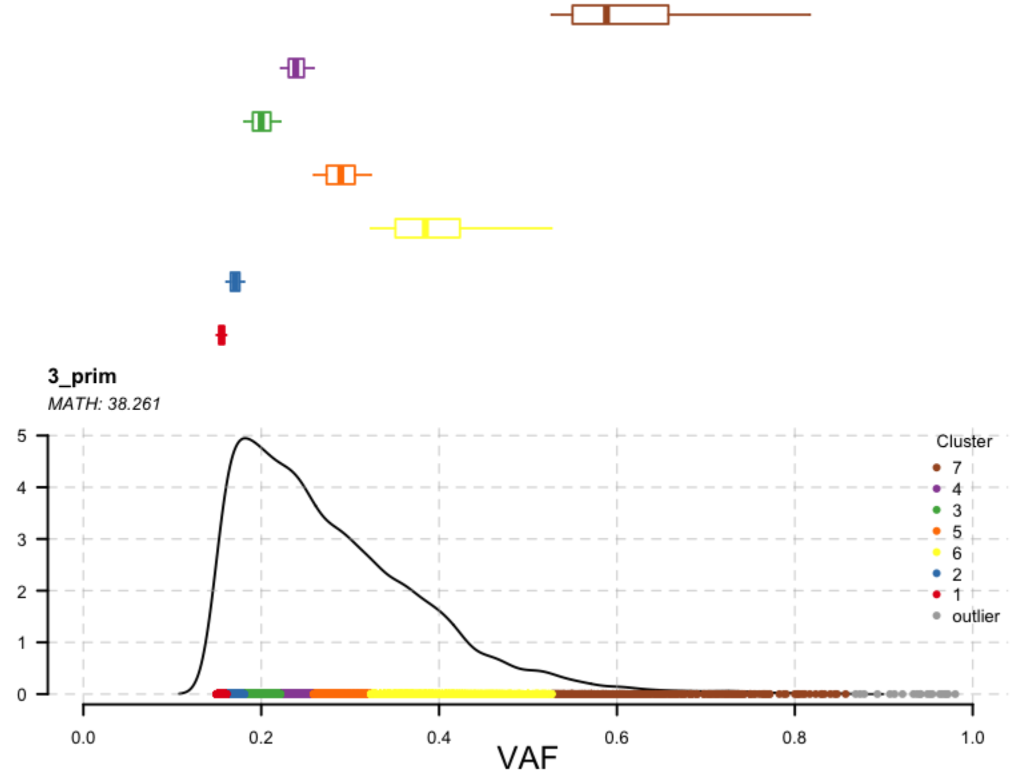

B

GBM3 R

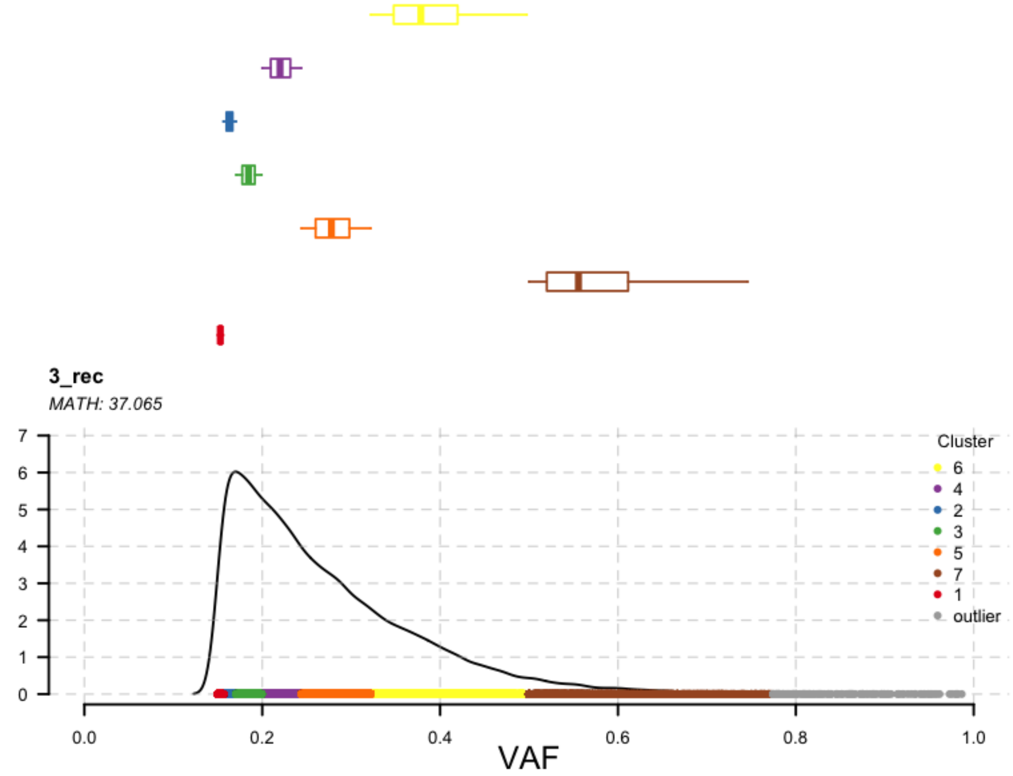

C

cluster	meanVaf	Min	25perc	Median	75perc	Max	No. MUTs	No. Genes
7	0.6197760	0.532995	0.5572600	0.600000	0.666667	0.857143	376	314
4	0.2392191	0.222222	0.2305160	0.238636	0.247572	0.258216	2043	1363
3	0.2006917	0.181034	0.1904760	0.200000	0.210526	0.221557	2590	1594
5	0.2918144	0.258427	0.2739730	0.290648	0.309091	0.328671	2954	1903
6	0.3986109	0.328767	0.3571430	0.388060	0.428571	0.532258	3278	2126
2	0.1708190	0.160804	0.1656440	0.170732	0.175676	0.180723	1363	910
1	0.1554303	0.150000	0.1526720	0.155556	0.158192	0.160622	781	558
outlier	0.9341280	0.868421	0.9095325	0.941176	0.964240	0.980392	20	19

D

cluster	meanVaf	Min	25perc	Median	75perc	Max	No. MUTs	No. Genes
6	0.3877576	0.323529	0.349206	0.3793100	0.420598	0.497512	19699	8372
4	0.2212955	0.200000	0.209790	0.2205320	0.232558	0.245455	20427	8619
2	0.1633662	0.156250	0.159722	0.1632650	0.166667	0.170648	8639	4761
3	0.1846619	0.170732	0.177515	0.1846150	0.191667	0.199313	15327	7237
5	0.2811721	0.245509	0.261905	0.2795700	0.299401	0.323353	22401	9200
7	0.5740460	0.500000	0.520000	0.5555560	0.611470	0.772727	3768	2429
1	0.1529481	0.150000	0.151515	0.1528380	0.154472	0.156146	3917	2563
outlier	0.8468322	0.774194	0.797297	0.8341485	0.886792	0.986486	166	131

A

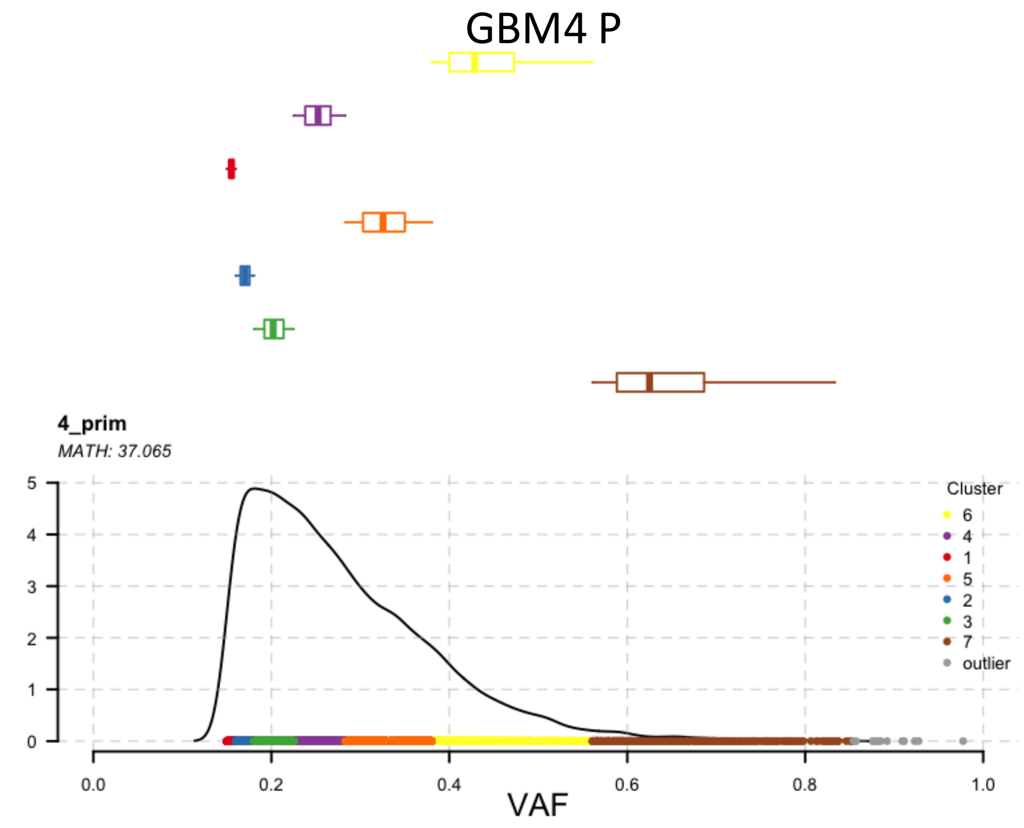

B

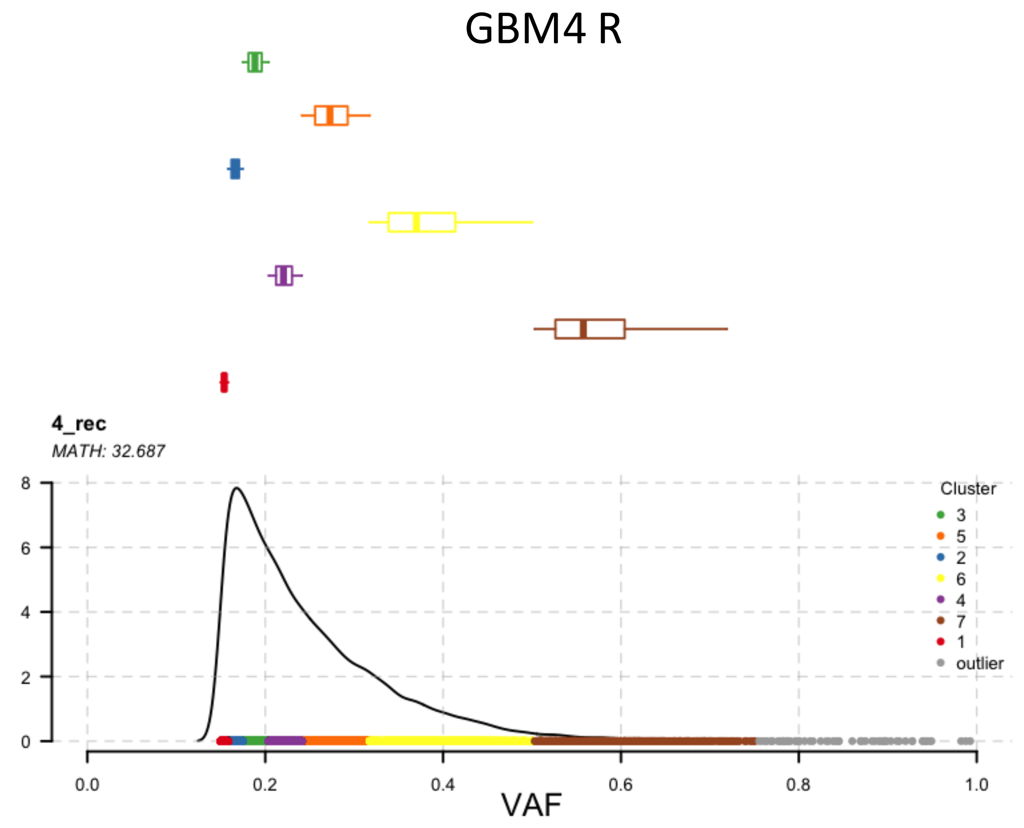

C

cluster	meanVaf	Min	25perc	Median	75perc	Max	No. MUTs	No. Genes
6	0.4414636	0.381818	0.400000	0.430769	0.473684	0.561644	3600	2347
4	0.2545749	0.228571	0.240964	0.253623	0.268041	0.283019	5721	3367
5	0.3277113	0.283186	0.303030	0.326531	0.350877	0.381679	6436	3704
1	0.1551726	0.150000	0.152672	0.155172	0.157895	0.160000	1320	915
2	0.1705213	0.160194	0.165414	0.170588	0.175573	0.180995	2729	1732
3	0.2043982	0.181034	0.192814	0.204082	0.216216	0.228395	5903	3319
7	0.6466910	0.562500	0.588235	0.625000	0.687500	0.857143	511	399
outlier	0.8996039	0.875969	0.879310	0.884615	0.911111	0.977413	13	12

D

cluster	meanVaf	Min	25perc	Median	75perc	Max	No. MUTs	No. Genes
3	0.1897041	0.176000	0.182482	0.189189	0.196581	0.204947	9476	5189
5	0.2770079	0.242604	0.257732	0.274725	0.295455	0.319588	11081	5841
2	0.1670098	0.158451	0.162602	0.166667	0.171429	0.175966	6929	4031
6	0.3826797	0.319767	0.342466	0.372881	0.415094	0.503497	8019	4595
4	0.2225658	0.204969	0.212963	0.222222	0.231579	0.242424	9070	5065
7	0.5730551	0.504854	0.526316	0.558233	0.604167	0.755396	909	718
1	0.1540799	0.150000	0.151832	0.154206	0.156250	0.158416	3614	2407
outlier	0.8512241	0.758621	0.786885	0.844289	0.896552	0.992701	70	41

GBM5 P

A

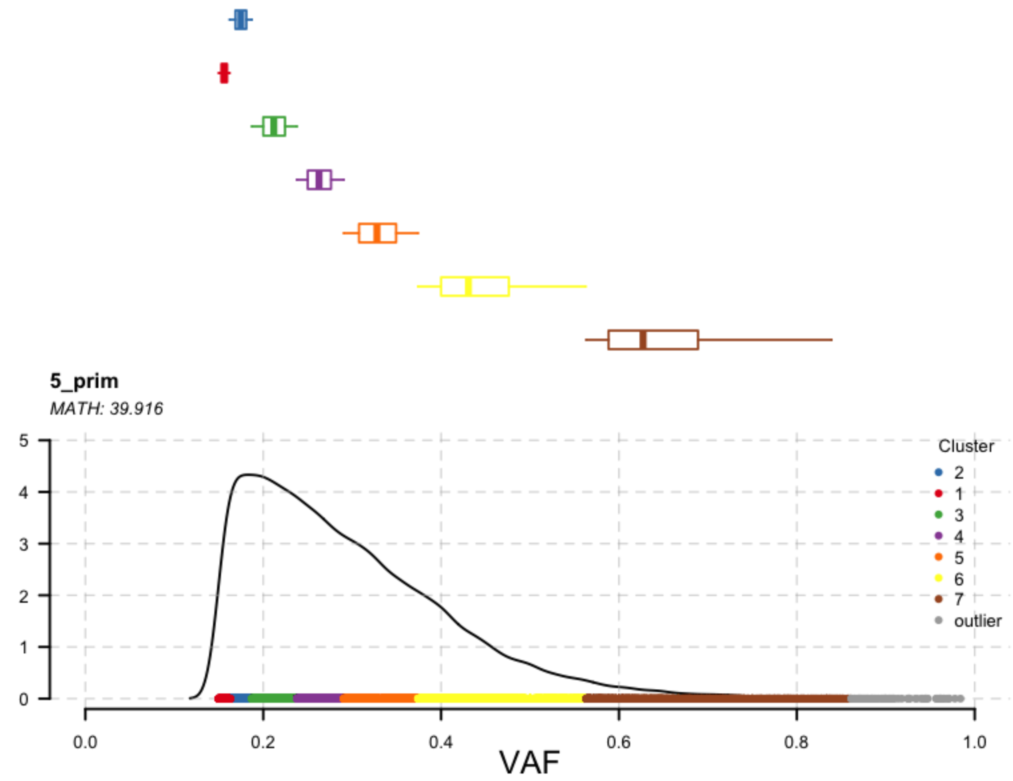

GBM5 R

B

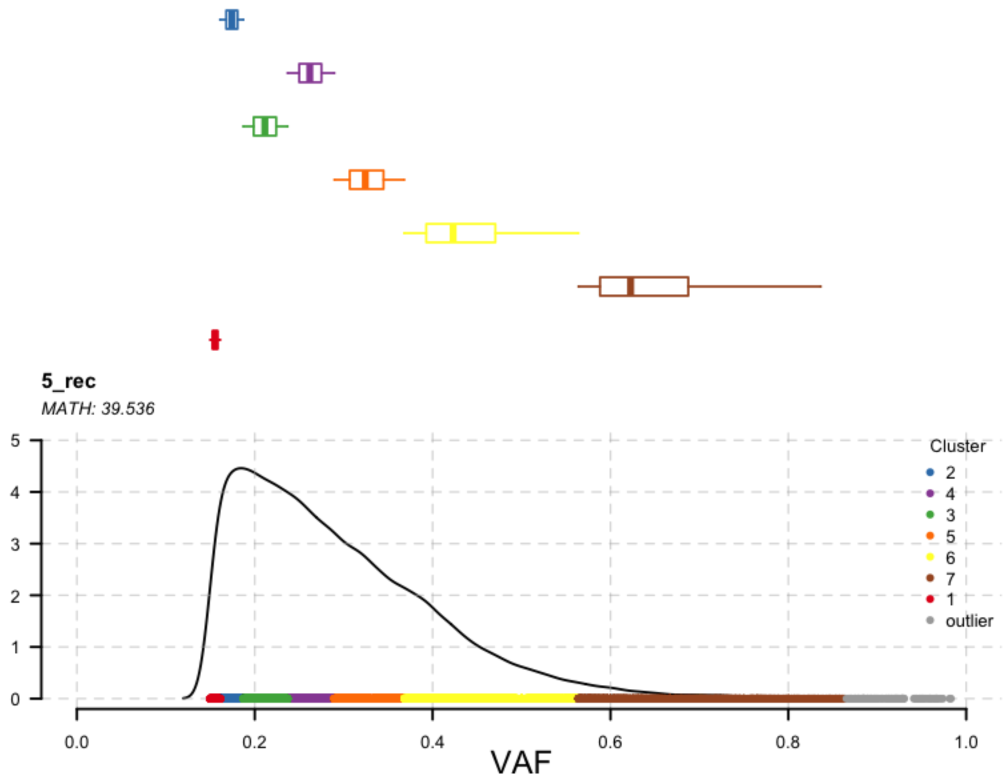

C

cluster	meanVaf	Min	25perc	Median	75perc	Max	No. MUTs	No. Genes
2	0.1749541	0.162281	0.168831	0.175000	0.1812500	0.187500	8676	4486
1	0.1561502	0.150000	0.153005	0.156250	0.1592360	0.162252	4357	2570
3	0.2119436	0.187661	0.200000	0.211679	0.2242990	0.237037	16303	7038
4	0.2648065	0.237113	0.250000	0.264000	0.2787745	0.294574	15908	7184
5	0.3341118	0.294643	0.312500	0.333333	0.3548390	0.379310	17129	7595
6	0.4462509	0.379630	0.403846	0.435294	0.4807690	0.567164	14233	6714
7	0.6529135	0.567568	0.595745	0.631579	0.6946795	0.864407	2308	1546
outlier	0.9126447	0.864865	0.883721	0.904110	0.9444440	0.984000	81	70

D

cluster	meanVaf	Min	25perc	Median	75perc	Max	No. MUTs	No. Genes
2	0.1745654	0.161194	0.1680000	0.174603	0.181102	0.187817	11395	5585
4	0.2651199	0.241379	0.2527470	0.264516	0.277778	0.290816	17197	7747
3	0.2138684	0.187845	0.2000000	0.213592	0.227273	0.241206	21796	8660
5	0.3255762	0.290909	0.3076920	0.323944	0.343750	0.364706	19032	8309
6	0.4326697	0.364865	0.3906250	0.421053	0.466667	0.560976	19921	8429
7	0.6415398	0.561404	0.5862070	0.619048	0.684211	0.860465	2621	1709
1	0.1553761	0.150000	0.1526720	0.155340	0.158228	0.161137	4671	2806
outlier	0.9143750	0.862745	0.8849305	0.913043	0.942286	0.982759	107	81

A

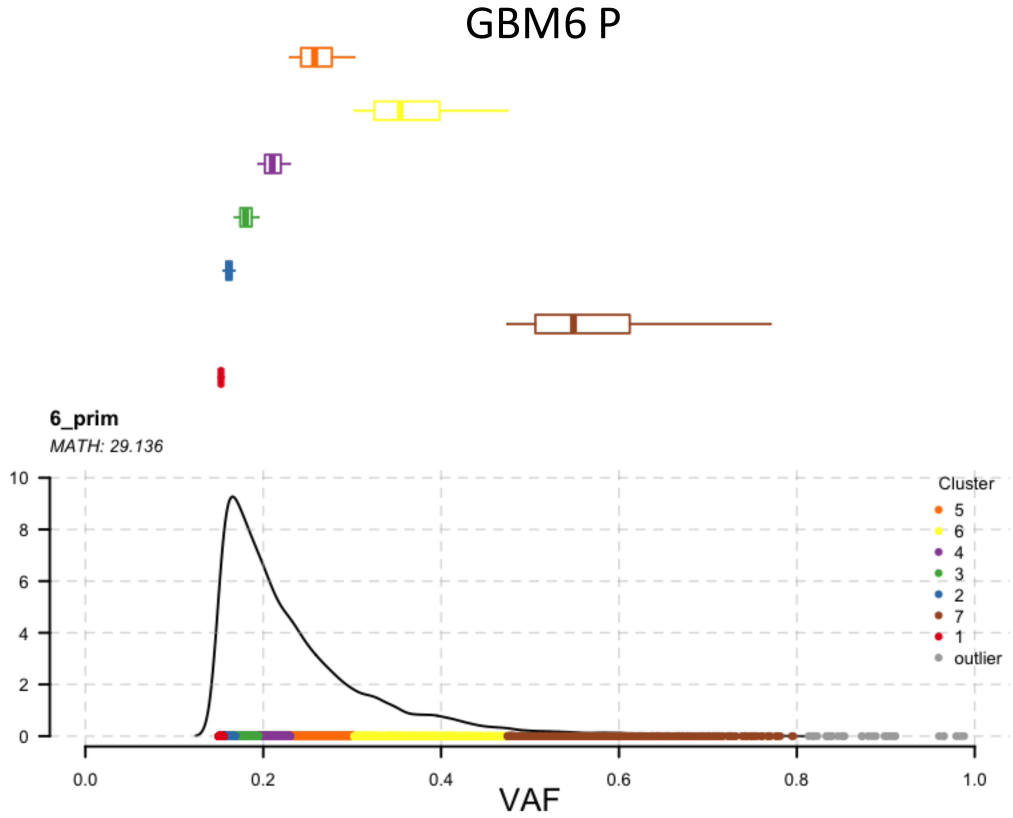

B

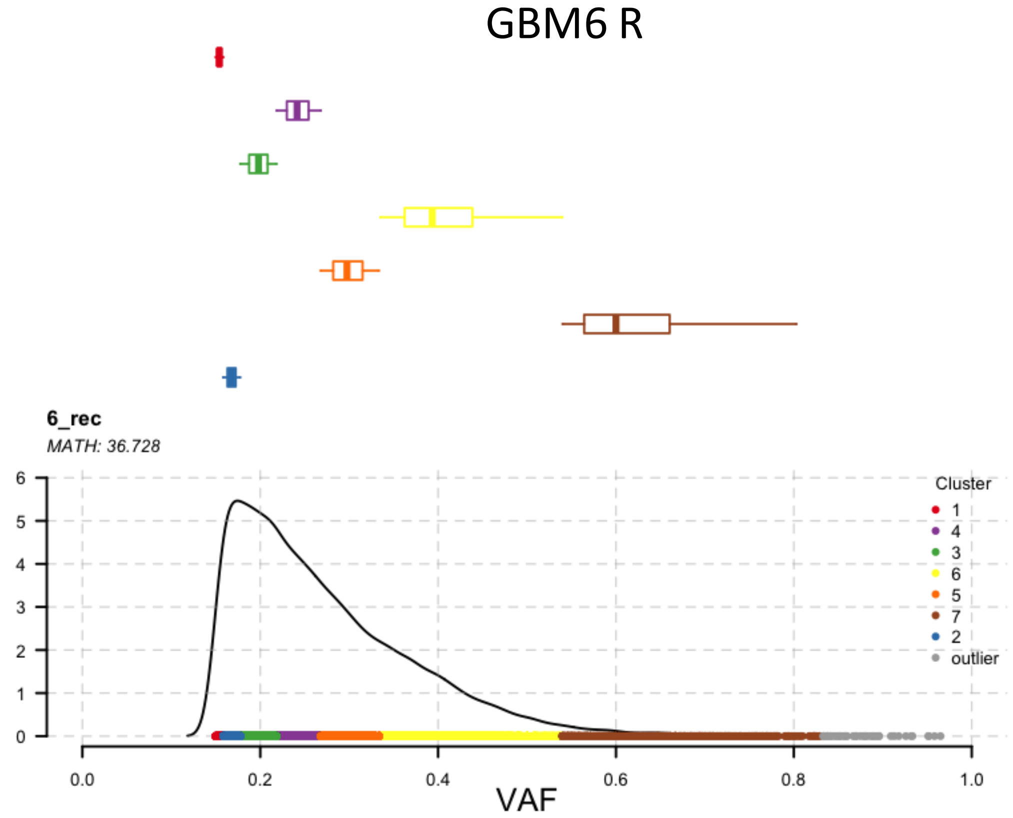

C

cluster	meanVaf	Min	25perc	Median	75perc	Max	No. MUTs	No. Genes
5	0.2523517	0.222222	0.234177	0.2500000	0.269231	0.294737	5494	3301
6	0.3573922	0.294872	0.318182	0.3473280	0.392157	0.466859	3641	2469
4	0.2039355	0.188976	0.196078	0.2033900	0.211538	0.221557	4496	2840
3	0.1771146	0.166667	0.171233	0.1769910	0.182609	0.188940	4450	2804
2	0.1602971	0.154930	0.157480	0.1602210	0.162963	0.166107	2593	1760
7	0.5613168	0.467480	0.500000	0.5410800	0.605505	0.781250	742	633
1	0.1522714	0.150000	0.151079	0.1522735	0.153409	0.154839	1308	944
outlier	0.8766663	0.794521	0.835165	0.8633375	0.907216	0.988095	34	31

D

cluster	meanVaf	Min	25perc	Median	75perc	Max	No. MUTs	No. Genes
1	0.1540671	0.150000	0.151832	0.153846	0.156250	0.158273	2258	1462
4	0.2435500	0.220126	0.231579	0.242991	0.255639	0.268966	8811	4671
3	0.1988273	0.177866	0.188406	0.198347	0.209677	0.220065	9761	4806
6	0.4083045	0.339450	0.366197	0.397436	0.441558	0.543478	8999	4876
5	0.3012511	0.269231	0.283784	0.300000	0.318182	0.339286	8706	4719
7	0.6261624	0.543860	0.568627	0.602740	0.666667	0.837838	861	682
2	0.1680620	0.158416	0.163121	0.168000	0.172662	0.177778	4844	2752
outlier	0.8852148	0.840909	0.857143	0.880000	0.902462	0.964912	39	38

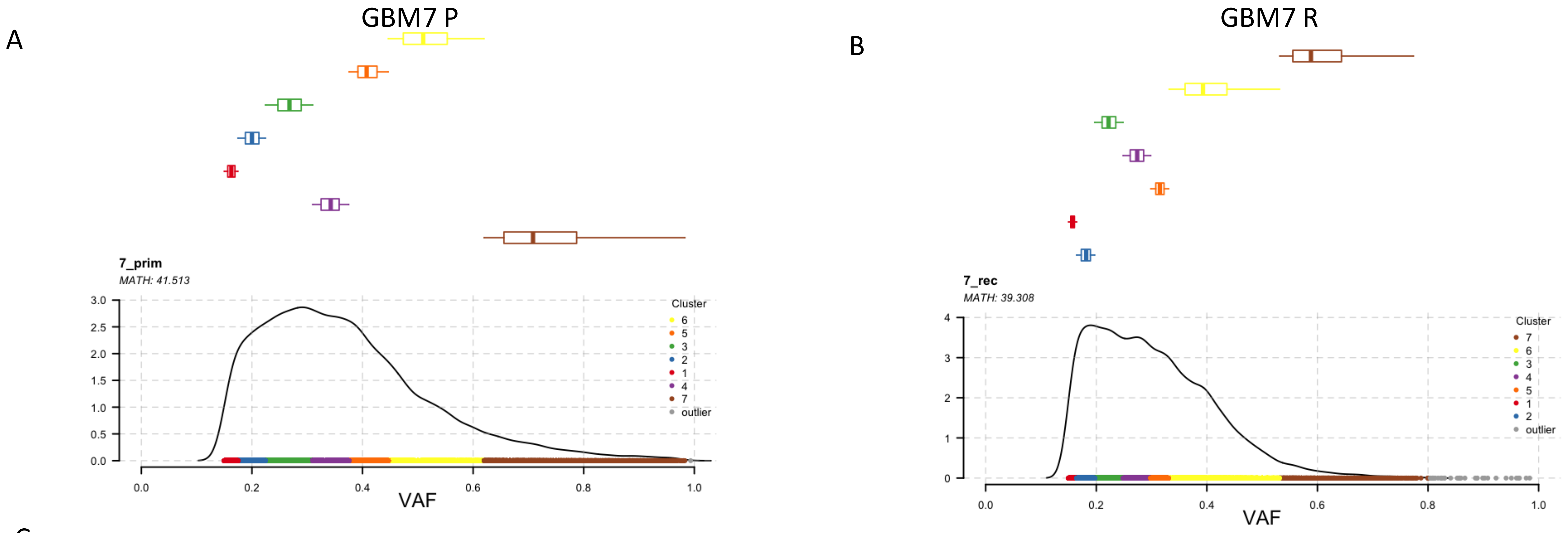

C

cluster	meanVaf	Min	25perc	Median	75perc	Max	No. MUTs	No. Genes
5	0.4925585	0.418079	0.4468090	0.4827590	0.5333330	0.606061	9286	4693
3	0.2725871	0.225806	0.2500000	0.2727270	0.2954550	0.317757	10338	4461
4	0.3663264	0.317829	0.3424660	0.3659335	0.3906250	0.417910	10270	4885
2	0.2012784	0.175497	0.1889760	0.2018350	0.2135920	0.225641	4768	2221
1	0.1630871	0.150000	0.1562500	0.1632650	0.1698110	0.175439	2130	1058
6	0.7209399	0.606557	0.6458330	0.6981130	0.7777780	0.975000	3147	1927
outlier	0.9816603	0.977273	0.9786365	0.9805660	0.9820915	0.993421	8	6

D

cluster	meanVaf	Min	25perc	Median	75perc	Max	No. MUTs	No. Genes
7	0.6105034	0.534247	0.557692	0.590164	0.648886	0.809524	688	513
6	0.4029199	0.333333	0.360656	0.393443	0.437500	0.533333	6861	3737
3	0.2240231	0.197080	0.210526	0.224138	0.236842	0.252336	4200	2263
5	0.3131395	0.295082	0.304348	0.313433	0.322581	0.331776	2345	1508
4	0.2737099	0.252427	0.263158	0.273973	0.285714	0.294964	3259	1931
1	0.1572534	0.150000	0.153846	0.157480	0.160584	0.164502	1230	717
2	0.1811800	0.164557	0.172886	0.181818	0.189189	0.196970	2635	1467
outlier	0.8819922	0.812500	0.829787	0.863021	0.923077	0.983871	30	26

A

GBM8 P

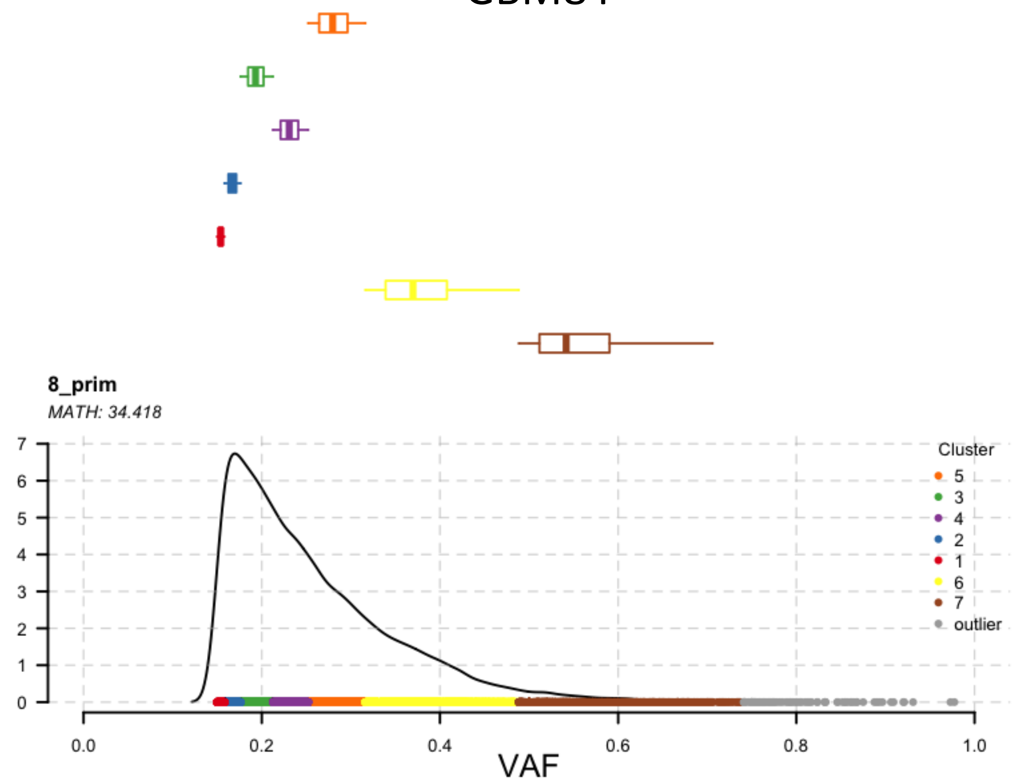

B

GBM8 R

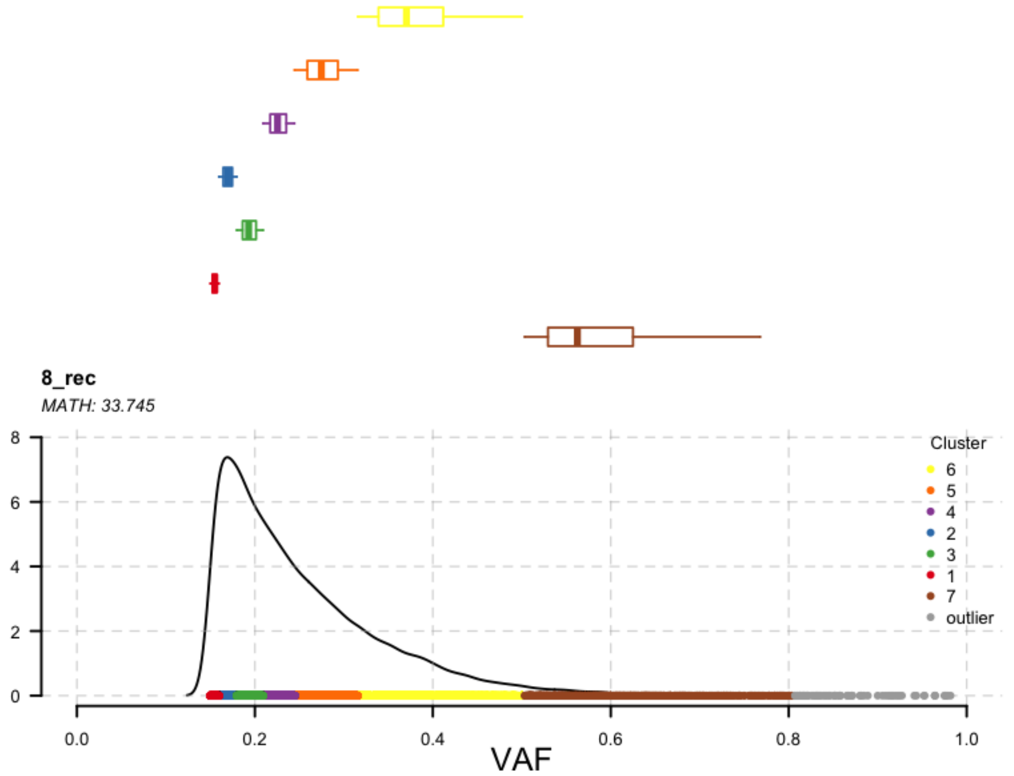

C

cluster	meanVaf	Min	25perc	Median	75perc	Max	No. MUTs	No. Genes
5	0.2800628	0.252033	0.2638465	0.278846	0.294872	0.313471	7811	4505
3	0.1933490	0.176000	0.1842110	0.192982	0.201923	0.212389	8882	4915
4	0.2310162	0.212435	0.2210530	0.230769	0.240964	0.251969	7383	4334
1	0.1539325	0.150000	0.1518990	0.153846	0.156028	0.157895	2450	1712
6	0.3755202	0.313559	0.3382350	0.368421	0.406780	0.486111	8052	4660
2	0.1668049	0.158088	0.1623380	0.166667	0.171053	0.175966	5081	3140
7	0.5569340	0.486486	0.5106380	0.540541	0.590361	0.741573	1153	916
outlier	0.8120960	0.741935	0.7617910	0.796296	0.854715	0.977359	75	64

D

cluster	meanVaf	Min	25perc	Median	75perc	Max	No. MUTs	No. Genes
6	0.3812357	0.317308	0.340659	0.370968	0.412698	0.503650	9922	5382
5	0.2809724	0.250965	0.264706	0.279412	0.296296	0.317073	10360	5565
4	0.2300567	0.211268	0.220000	0.229358	0.240000	0.250000	9873	5311
2	0.1700099	0.159763	0.164894	0.169857	0.175182	0.180412	8374	4564
3	0.1950250	0.180451	0.187050	0.194690	0.202703	0.211180	10286	5414
1	0.1548410	0.150000	0.152318	0.155000	0.157427	0.159722	4371	2745
7	0.5861361	0.504065	0.529412	0.563380	0.625000	0.803571	1267	989
outlier	0.8711947	0.807018	0.826087	0.857143	0.913793	0.981818	74	64

A

B

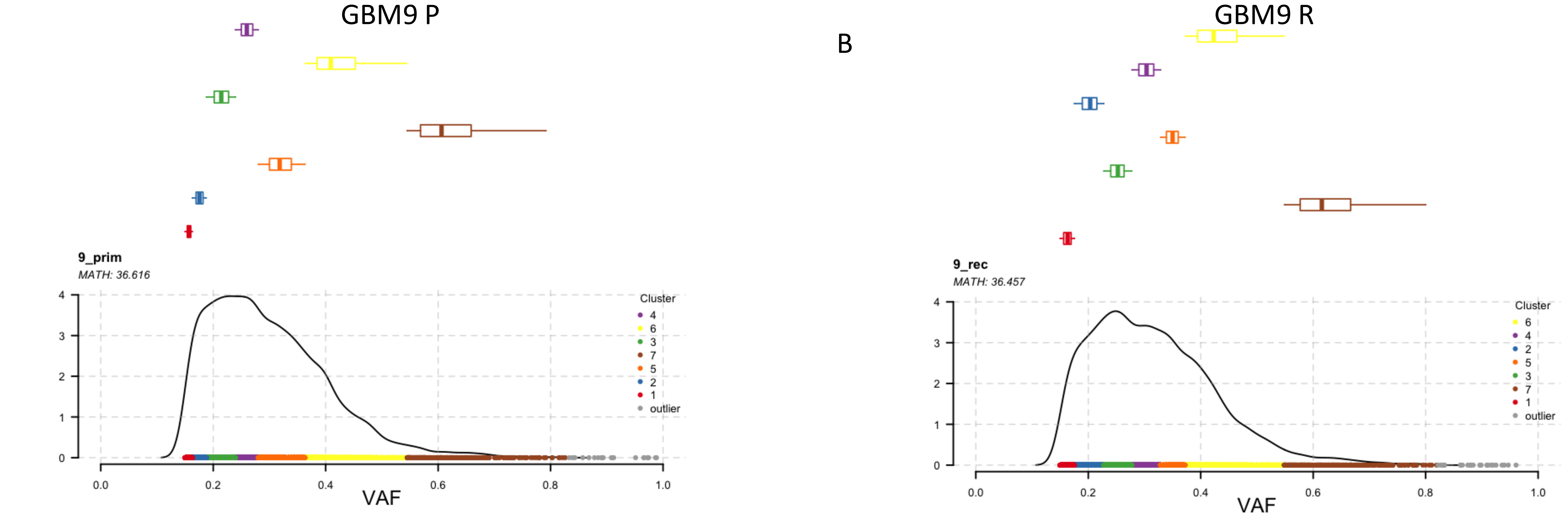

C

cluster	meanVaf	Min	25perc	Median	75perc	Max	No. MUTs	No. Genes
4	0.2641043	0.241935	0.252874	0.264368	0.274725	0.287037	2345	1514
6	0.4306839	0.371795	0.393443	0.420000	0.461538	0.549020	2622	1738
5	0.3275455	0.287234	0.306667	0.327869	0.348400	0.371429	3523	2193
3	0.2147948	0.186747	0.201613	0.215054	0.228261	0.241758	2923	1809
7	0.6271295	0.550000	0.574074	0.615385	0.663934	0.826087	338	279
2	0.1748450	0.162791	0.169231	0.174603	0.180328	0.186667	1171	792
1	0.1567445	0.150000	0.153846	0.156977	0.160000	0.162651	581	424
outlier	0.9136194	0.833333	0.878049	0.908046	0.966102	0.988372	21	18

D

cluster	meanVaf	Min	25perc	Median	75perc	Max	No. MUTs	No. Genes
6	0.4137564	0.344828	0.3728810	0.4035090	0.4444440	0.544118	4817	2786
4	0.3023605	0.278846	0.2906980	0.3030300	0.3132530	0.325000	2345	1493
2	0.2024101	0.175325	0.1893940	0.2033900	0.2156860	0.227723	2536	1505
5	0.3351926	0.325203	0.3290305	0.3333330	0.3389830	0.344538	959	669
3	0.2530664	0.227848	0.2409640	0.2530120	0.2658230	0.278481	2885	1708
7	0.6241974	0.544304	0.5714290	0.6115255	0.6644145	0.810811	516	381
1	0.1629790	0.150000	0.1562500	0.1632650	0.1694920	0.175182	968	608
outlier	0.8840150	0.819444	0.8477010	0.8823075	0.9138890	0.960784	28	23

A

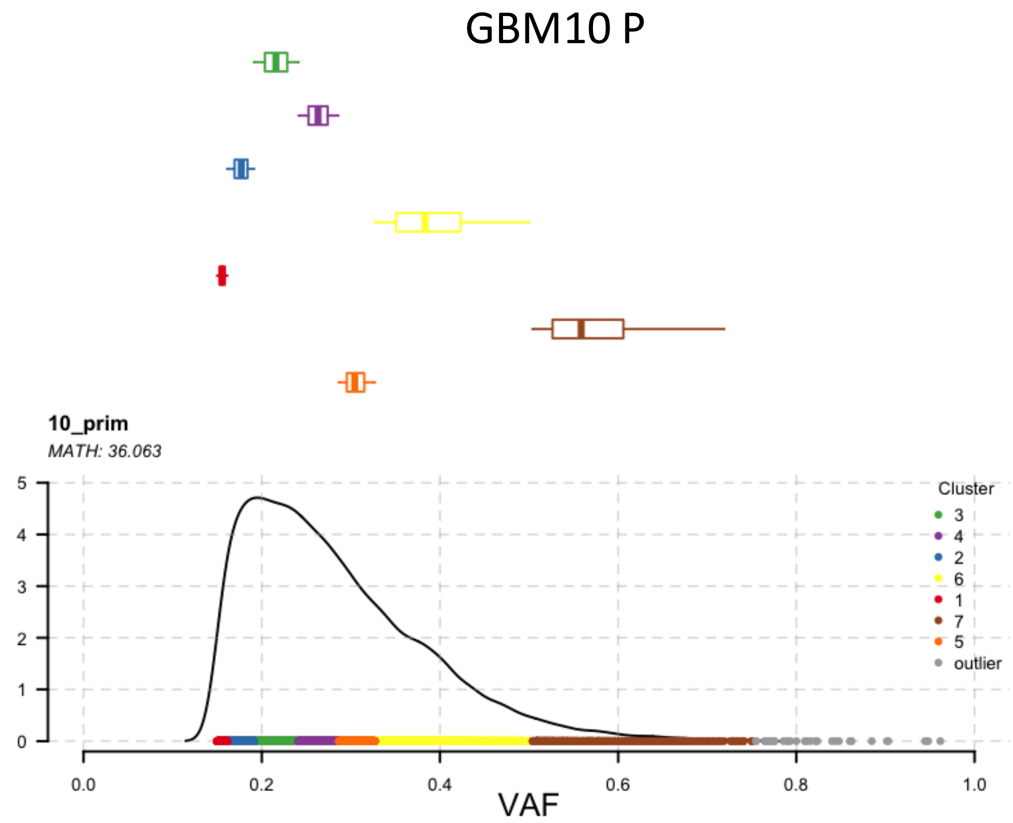

B

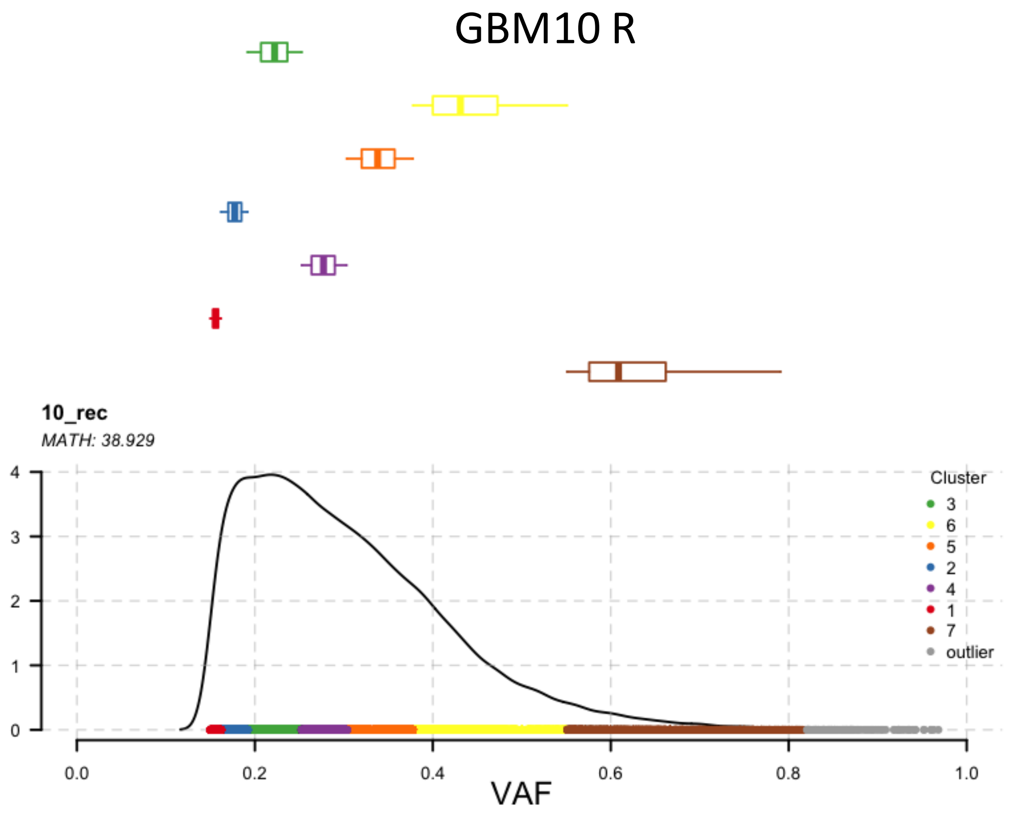

C

cluster	meanVaf	Min	25perc	Median	75perc	Max	No. MUTs	No. Genes
3	0.2151217	0.190840	0.202073	0.215054	0.2272730	0.239726	5577	3265
4	0.2624123	0.240000	0.250000	0.261905	0.2739730	0.285714	4610	2869
2	0.1765488	0.161290	0.169492	0.176923	0.1834860	0.190789	3279	2082
6	0.3945437	0.330986	0.357143	0.385965	0.4262300	0.504000	5812	3495
1	0.1555970	0.150000	0.152866	0.155556	0.1582730	0.161157	1145	811
7	0.5739865	0.504673	0.526316	0.558824	0.6060610	0.750000	842	652
5	0.3075910	0.286517	0.296296	0.307692	0.3181820	0.330579	3287	2156
outlier	0.8167720	0.754717	0.771353	0.793103	0.8454815	0.961538	35	31

D

cluster	meanVaf	Min	25perc	Median	75perc	Max	No. MUTs	No. Genes
3	0.2214065	0.191781	0.2066120	0.2212390	0.235955	0.250000	14503	6396
6	0.4433047	0.381250	0.4038460	0.4333330	0.476190	0.553191	11774	5992
5	0.3408614	0.305263	0.3222220	0.3389830	0.359375	0.380952	12725	6177
2	0.1772823	0.161932	0.1700015	0.1774190	0.184874	0.191667	7047	3753
4	0.2774238	0.251142	0.2631580	0.2772280	0.290323	0.305085	11510	5587
1	0.1559916	0.150000	0.1530050	0.1560280	0.158824	0.161905	2525	1580
7	0.6292064	0.553571	0.5769230	0.6111110	0.666667	0.824561	2235	1582
outlier	0.8770254	0.827586	0.8461540	0.8686365	0.904753	0.968254	96	79

Figure S1. Tumor heterogeneity and clonality. The figures depict tumor heterogeneity inferred by clustering VAFs of all detected variants (except first-level variants in Table 5) in our primary (A) and recurrent (B) sample pairs. Clusters are separated based on the mean VAFs using mclust. The x axis shows the allele frequency on the scale 0–1, and the y axis shows the variant density within genes. The MATH score (mentioned as a subtitle in the plot) is a simple quantitative measure of intra-tumor heterogeneity, which calculates the width of the vaf distribution. Higher MATH scores are found to be associated with more heterogenous samples. The distribution of variant allele frequencies within a cluster are represented as boxplots above the line graphs. The tables (C and D) show the corresponding VAF numbers in detail (mean, minimum, max, 25th and 75th percentiles) supplemented by the number of mutations and genes in each cluster in the current primary and recurrent sample pairs.
